# Trees and networks before and after Darwin

**DOI:** 10.1186/1745-6150-4-43

**Published:** 2009-11-16

**Authors:** Mark A Ragan

**Affiliations:** 1 The University of Queensland, Institute for Molecular Bioscience and Australian Research Council Centre of Excellence in Bioinformatics, 306 Carmody Rd, St Lucia, Brisbane, Queensland 4072, Australia

## Abstract

It is well-known that Charles Darwin sketched abstract trees of relationship in his 1837 notebook, and depicted a tree in the *Origin of Species *(1859). Here I attempt to place Darwin's trees in historical context. By the mid-Eighteenth century the Great Chain of Being was increasingly seen to be an inadequate description of order in nature, and by about 1780 it had been largely abandoned without a satisfactory alternative having been agreed upon. In 1750 Donati described aquatic and terrestrial organisms as forming a network, and a few years later Buffon depicted a network of genealogical relationships among breeds of dogs. In 1764 Bonnet asked whether the Chain might actually branch at certain points, and in 1766 Pallas proposed that the gradations among organisms resemble a tree with a compound trunk, perhaps not unlike the tree of animal life later depicted by Eichwald. Other trees were presented by Augier in 1801 and by Lamarck in 1809 and 1815, the latter two assuming a transmutation of species over time. Elaborate networks of affinities among plants and among animals were depicted in the late Eighteenth and very early Nineteenth centuries. In the two decades immediately prior to 1837, so-called affinities and/or analogies among organisms were represented by diverse geometric figures. Series of plant and animal fossils in successive geological strata were represented as trees in a popular textbook from 1840, while in 1858 Bronn presented a system of animals, as evidenced by the fossil record, in a form of a tree. Darwin's 1859 tree and its subsequent elaborations by Haeckel came to be accepted in many but not all areas of biological sciences, while network diagrams were used in others. Beginning in the early 1960s trees were inferred from protein and nucleic acid sequences, but networks were re-introduced in the mid-1990s to represent lateral genetic transfer, increasingly regarded as a fundamental mode of evolution at least for bacteria and archaea. In historical context, then, the Network of Life preceded the Tree of Life and might again supersede it.

**Reviewers:**

This article was reviewed by Eric Bapteste, Patrick Forterre and Dan Graur.

## Prefatory quotation

*(N)ature rises up by connections, little by little and without leaps, as though it proceeds by an unbroken web, it proceeds in a leisurely and placid uninterrupted course. There is no gap, no break, no dispersion of forms: they have, in turn, been connected, ring within ring. That very golden chain is universal in its embrace. - *Juan Eusebio Nieremberg, 1635 [[1], p.29]

## The rise and fall of the Great Chain of Being

From very early in the Middle Eastern and European religious and intellectual traditions, chains, cords, ladders and stairways served as metaphors for order in the world, or between earth and heaven [2-6]. The image of a tree sometimes served in the same metaphorical sense [[5], pp.319-329; [6], p.22]. A linear order in nature was compatible, for example, with the hierarchical arrangement of creation implied by emanationist cosmology, correspondences between spiritual and earthly bodies, and the literal or figurative ascent of the soul or mind toward God. Even the incipient change, beginning in the Twelfth century, from a God-centric to a man-centric hierarchy provided no cause to question the underlying assumption of a linear arrangement or ordering in nature.

**Figure 1:**
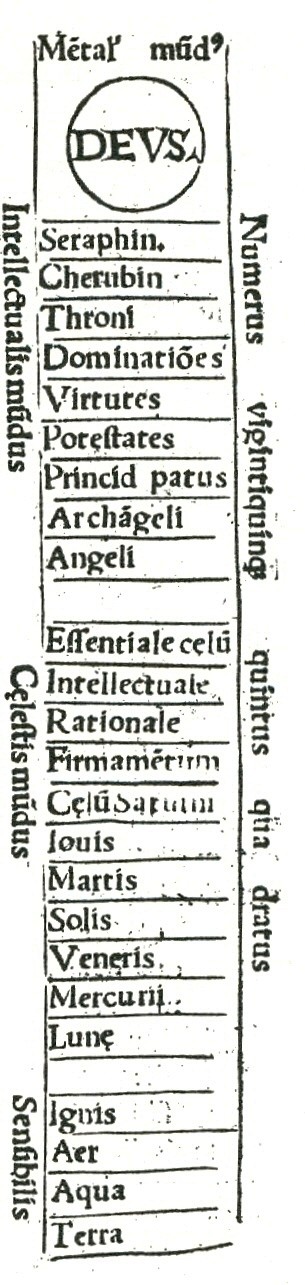
**Linear order in nature, from the *Physicorum elementorum *of Charles de Bouelles (1512)**. Organised bodies (*i.e*. minerals, vegetables, animals and man) are not explicitly placed within this chain.

**Figure 2:**
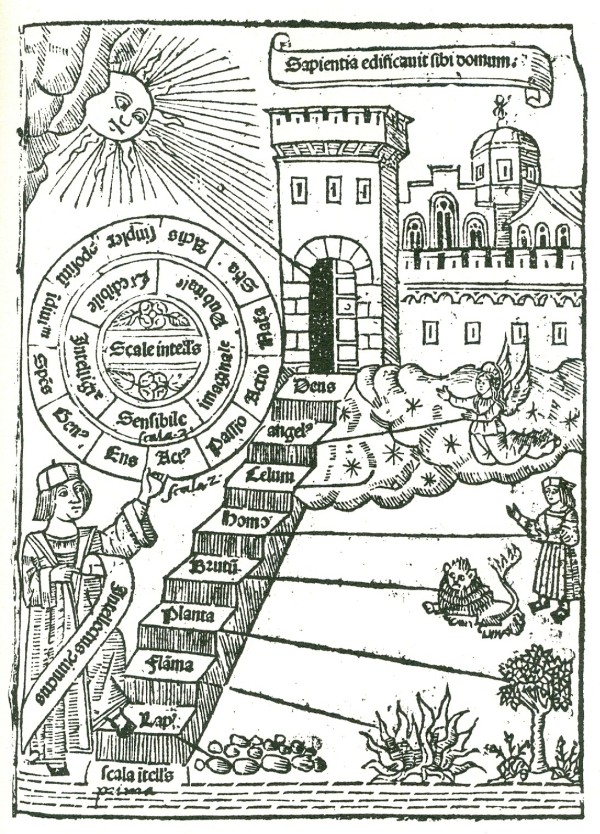
**Three scales of intellect connecting earth with the abode of God, from the *Liber de ascensu et decensu intellectus *of Ramon Llull (written 1304, first published 1512)**. Organised bodies (unusually including fire) are included in the main scale, depicted as a stairway.

Within this Great Chain of Being, organised matter on earth might constitute a greater or lesser portion. In the *Physicorum elementorum *of Charles de Bouelles [7], for example, earth, water, air and fire constituted only four of the 25 hierarchical levels that culminate in the Deity (Figure 1). By contrast, the *Liber de ascensu et descensu intellectus *of Ramon Llull [8] presents an eight-step stairway of which stones, fire, plants, brute animals and man form the first five steps, and the Deity is reached at step eight (Figure 2). In principle, not only large inclusive groups (animals, plants) but indeed every taxon of being, no matter how minor, might be ordered linearly, in accord with Aristotle's famous description of organized matter as

...*proceeding little by little from things lifeless to animal life in such a way that it is impossible to determine the exact line of demarcation, nor on which side thereof an intermediate form should lie. Thus, next after lifeless things in the upward scale comes the plant, and of plants one will differ from another as to its amount of apparent vitality; and, in a word, the whole genus of plants, whilst it is devoid of life as compared with an animal, is endowed with life as compared with other corporeal entities. Indeed, as we have just remarked, there is observed in plants a continuous scale of ascent toward the animal *[[9], 588b:4).

Beginning in the Sixteenth century, botanical and zoological treatises were sometimes arranged to present organisms as forming a more-or-less ascending (or descending) series [10-13]. A particularly fine-grained linear arrangement appeared in the 1788 *Flore Françoise *by Jean-Baptiste Lamarck [14], although Peter Stevens hints that this arrangement *per se *may be due more to the Abbé René-Just de Haüy than to Lamarck himself [[15], 79f145]. But already by that point in the late Eighteenth century, the Great Chain was widely seen as inadequate in describing biological diversity. This crisis was brought about by four related developments, which I briefly present in turn.

First (chronologically) was Richard Bradley's argument that degree of perfection must not be argued from mere Platonic form or essence, but arrived at instead by analysis of "figure or parts" [[16], p.18]. His wonderful *Philosophical Account of the Works of Nature *(1721) arranges minerals, plants and animals in an ascending, if idiosyncratic, scale of nature, with innumerable descriptions, comparisons and analogies relating to their exterior body parts, internal circulation of fluids, features of reproduction, rates of growth and development, metamorphosis, activity, strength, "spirit" [[16], p.89] and "uses". Bradley was comfortable with the idea of *perfection *(*e.g*. of body parts), but observed multiple, non-uniform progressions, with individual species being imperfect in some regards and more-perfect in others. Indeed in respect of the linear arrangement of animals, Bradley famously concluded:

*"I suppose it may be wonder'd at, that hitherto I have not mentioned Mankind, who is so remarkable a Creature, and Lord of all the rest; I confess, was I to have placed him where the Parts of his Body would most agree with those of the created Bodies mention'd in this Treatise, I must have set him in the middle of this Chapter; but I suppose my Reader will excuse me, if I shew him so much regard, that I rather speak of him in the summing up of my Scale, than let him be encompass'd with wild Beasts." *[[16], p.117]

Second, the vast expansion in knowledge of animal, plant and microbial diversity in the Seventeenth and Eighteenth centuries [17-22] did not yield a more-continuous Chain; critics from Voltaire [[23], pp.58-60] to Dr Johnson [24] (and later, Eldridge & Gould [25]) could point to innumerable discontinuities, not to mention the fundamental paradox of continuity among discrete entities. Churchmen and philosophers had long grappled with the concept of plenitude (complete realisation of possibility) given the obvious imperfection of the world [4], with Peter Lombard arguing that we cannot deny to God the fullest scope of action in creation, *i.e*. he could have created a more-complete, more-perfect universe had he so willed [26]. But had the voyages of discovery been set up as an experimental test of morphological continuity across nature, the null hypothesis (discontinuity) would not have been rejected.

Third, and far more seriously, certain high-profile discoveries could not be fit into any reasonable linear arrangement of nature. Debate raged through the first half of the Eighteenth century (and beyond) on whether coral polyps were animal, vegetable, mineral, or some combination thereof [16,27-30]. Placing the green *Hydra *[31] among the mosses, for instance, would remove it far from the self-motile worms or insects. Likewise it seemed impossible to decide whether "the little Proteus" *Volvox *[[32], III pp.621-624] should be placed at the base of the plant scale, or at the base of the animals. In his beautifully written *Contemplation de la Nature *(1764-65), sometimes considered the culmination of the case for a Great Chain of Being, Charles Bonnet felt compelled to ask whether insects and shellfish need to be assigned to "lateral and parallel branches off this great Trunk" [[33], III p.xx].

Finally, it was becoming increasingly apparent that the continuity, if any, between plants and animals did not join the most-perfect plants (for Bonnet, "sensitive" plants such as *Mimosa*) with the least-perfect animals such as jellyfish. According to Linnaeus in aphorism 153 of *Philosophia botanica*:

*Nature herself associates and joins Minerals and Plants and Animals; but in so doing it does not connect the most perfect Plants with Animals that are said to be the most imperfect, but imperfect Animals and imperfect Plants are combined*.... [[34], §153]

I consider it unlikely that Linnaeus intended to argue that the combining (*combinat*) of imperfect animals and imperfect plants was more-integral than the associating and joining (*sociat et conjugit*) of minerals, plants and animals, and not only because *sociat *is equally well translated *unites*. Others later did draw this distinction: Flittner [35], for example, claimed that zoophytes, corals, sponges and seaweeds shared both plant and animal natures; and dual or alternating identities were frequently invoked *e.g*. in descriptions of coral polyps, alternating life-history stages of algae, and unicellular organisms such as diatoms [36,37]. In aphorism 153 Linnaeus probably did, however, intend to contrast "imperfect" with "maximally imperfect": nature joined the former.

Of course, linking the most-simple plants with the most-simple animals would yield not a linear scale or chain, but rather a dichotomy, as in the letter V; and if plants and animals were contiguous at multiple points (not only the maximally imperfect), order in nature would more resemble the letter Y. The Great Chain was in deep crisis.

## The search for alternative descriptions of nature

Although at first Linnaeus accepted that nature is ordered in a linear scale [38], by 1750 or 1751 he realized that even the plants could not be arranged in a simple unitary continuum. This casts an interesting light on aphorism 77 of his *Philosophia Botanica *[[34], §77], the three well-known parts of which might not, on initial reading, seem to be closely related:

*The fragments of the natural method are to be diligently sought out. This is the first and last desideratum in botanical study. Nature does not make leaps. All plants show affinities on either side, like territories in a geographical map*.

In the first two sentences, Linnaeus acknowledges that his own system of nature corresponds to the natural method only in small disjoint parts (fragments). Nature is, however, without gaps (Linnaeus takes this quotation verbatim from Ray [39]) and among the plants one family can be contiguous to one, two, or more than two others - unlike in a chain, where each family must have exactly two neighbors (even the least-perfect plants would be joined below to the minerals, as the most-perfect plants would be joined above to the animals). His map analogy seems not to have been taken literally at first, as no Map of Plants appears to have been rendered until Paul Giseke did so in 1792, fourteen years after Linnaeus's death [40]; but thereafter the cartographic enterprise persisted until 1859 and beyond, with Alphonse de Candolle developing rules for distributing plant taxa on a two-dimensional "map" [41]. Sachs [[42], p.137], Stevens [43], O'Hara [44] and Ragan [37] mention other early maps of nature.

The concept *affinity *bears further comment. In the late Eighteenth and very early Nineteenth centuries, affinities were regularities of resemblance or arrangement among characteristic or functionally important body parts (*e.g*. those constituting skeletal or organ systems) that indicated an attraction or closeness between the organisms or taxa in which they were found. Some authorities, observing the investigations of Lavoisier on describing the "affinities" that determine how chemical substances attract each other and form compounds of fixed stoichiometry, sought or imagined corresponding affinities in the biological realm that could likewise reveal the true order in nature, or natural laws [[45], pp.38-39 and chapter 8;[46], I pp.535-540]. These affinities could be "morphological, structural, and physiological" [[47], IV p.81]. Later, affinity came to be reserved for taxa, whereas the corresponding relation among characters would be called *homology *[[44], p.258]. Innate *affinity *was properly contrasted with *analogy*, which was external, superficial or remote.

## Trees before Darwin

**Figure 3:**
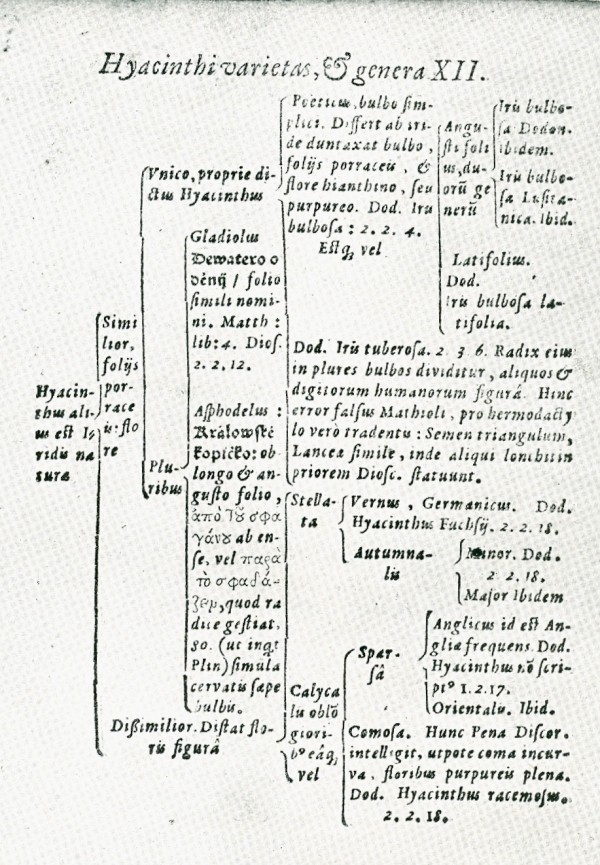
**Bifurcating key of hyacinth species, from the *Methodi herbariae libri tres *of Adam Zaluziansky à Zaluzian (1592)**.

Keys are hierarchical arrangements intended to aid identification or information retrieval, and are sometimes presented as a branching logical structure, *i.e*. a tree. An early key of plant genera, by Zaluziansky à Zaluzian (1592), is shown in Figure 3 [[12], opp. p.87], and keys were relatively commonplace by the second quarter of the Eighteenth century (*e.g*. Ray, van Royen, Linnaeus).

Much more interestingly, nearly a century before Darwin's *Origin of Species*, Peter Simon Pallas proposed in *Elenchus Zoophytorum *[48] that the gradation among organisms might best be described as a branching tree:

*"...various authors desire a certain pleasing scale in nature, of such excellence as will never be found, such as Bradley and Bonnet wish for. The gradation can be expressed no less well, indeed very much better, as various affinities in polyhedric figures, [with] genera of organic bodies distributed close by in numerous small spaces by turns. As Donati has already judiciously observed, the works of Nature are not connected in series in a Scale, but cohere in a Net. On the other hand, the whole system of organic bodies may be well represented by the likeness of a tree that immediately from the root divides both the simplest plants and animals, [but they remain] variously contiguous as they advance up the trunk, Animals and Vegetables; those leading, from Mollusca advancing to Pisces, with great lateral branches of Insects sent out among themselves, from here to Amphibia; and at the extreme top of the tree the Quadrupeds are supported, Aves truly thrust out as an equally great lateral branch below the Quadrupeds. At the same time this image shows the animals to be neither continuous nor neighboring, but standing like a lone tree. Its trunk is the series of the more principal neighboring genera closely appressed; everywhere genera are thrust out like twigs, yet [the principal genera] are never connected to each other by lateral relationships." *[[48], pp.23-24]

His key points are that (1) animals and vegetables separate from each other immediately at the base of the trunk and proceed upward on their own, forming a dichotomous trunk; (2) the animal and vegetable trunks nonetheless remain contiguous at various points; (3) the animal (and separately, the plant) trunk is formed from series of principal neighboring genera pressed closely against each other; (4) genera are thrust out everywhere "like twigs"; and (5) no lateral relationships connect the principal genera (in the plant trunk or in the animal trunk).

**Figure 4:**
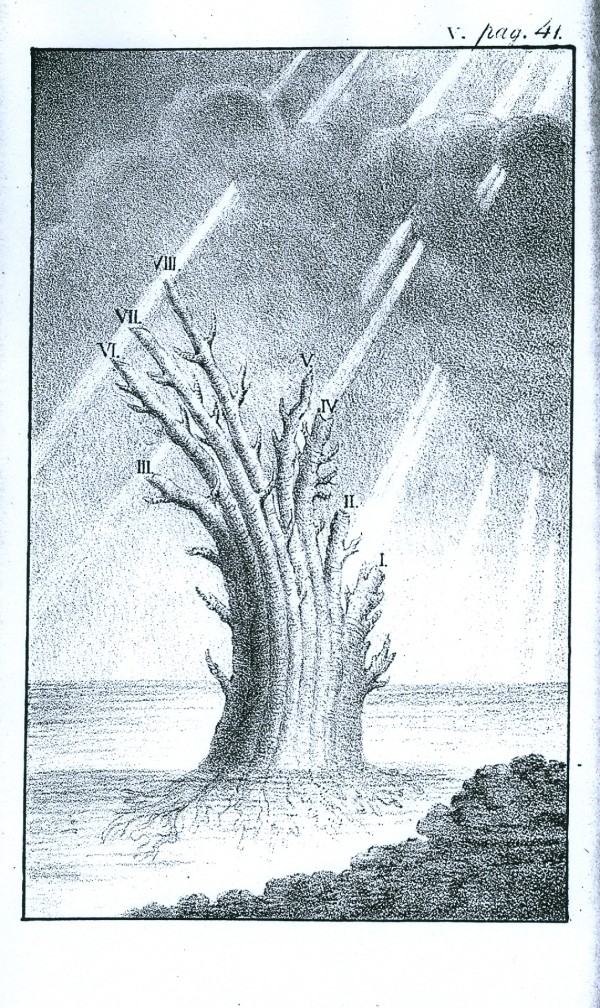
**Tree of animal life, from the *Zoologia specialis *of [Carl] Edward [von] Eichwald (1829)**. Historian Semyon Mikulinskii (1972) describes this as the first depiction of the tree proposed by Peter Simon Pallas in *Elenchus Zoophytorum *(1766).

Regarding the third of these points, Pallas wrote *Hac figura indicaretur simul Corpora organica, brutis non continua nec affinia esse, sed tantum insistere ceu arbor solo. Truncus e principaliori generum affinium serie confertus*. The word *principaliori *might be translated as *more original *or even *earlier*, but we must be careful not to impute a temporal dimension. A 1787 German translation [[49], p.48] rendered this passage *Der aus der vorzüglichern Reihe anverwandter und dicht für einander stehender Geschlechter zusammengesetzte Stamm*..., which in turn may be translated "The trunk, composed of the principal series of genera that are related and stand compact to one another...". Unfortunately Pallas himself is not known ever to have sketched his tree, but the sense conveyed is of a compound trunk - not the solid trunk of the much later interpretation by Thienemann [50], but instead a bunch of asparagus-shoots as in the "tree of animal life" of Eichwald (Figure 4) [51]. Interestingly, Mikulinskii [[52], p.344; [53], p.315] presents Eichwald's diagram as the first to have depicted Pallas's tree.

Although a prolific author, Carl Edward von Eichwald is little-known today outside Russian-language histories of science (*e.g*. [52,53]) in which his name is given as Eduard Ivanovich Eichwald. In his 1821 *De regni animalis limitibus *[54], Eichwald states that animalcula (protozoa) "have the rudiments of animal organization, from whence the series of more-perfect animals evolve in ever-elaborating grades and multifarous ramifications". He says repeatedly that animals and plants proceed from a common primordium (for him an organisational concept, not an early point in time), with the simple animals and simple plants proximate in this primordium. Eichwald developed this idea further in *Zoologia specialis *[51], proposing that the six primary animal types (Spondylozoa; Podozoa, Taxozoa and Heterozoa; Therozoa; Grammozoa; Cyclozoa; and Phytozoa) had originated in the primaeval shallow ocean; the first type arose from abundant "globules of primitive mucous", followed by the others in temporal succession, each a branch off from, and elevated in relation to, the previous type [[51], p.41]. *Zoologia specialis *is less overtly nature-philosophical than Eichwald's earlier *De regni animalis limitibus *and clearly presents a branching, transformationist view of the origin of biodiversity. According to Mikulinskii, however, Eichwald was "not particularly a Darwinian in his later writings".

Even earlier in 1801, Augustin Augier [55] represented plants on a multifurcating *Arbre botanique*, and considered this representation to apply equally to animals and minerals:

*"The order that I established among plants is found equally in the three kingdoms of nature, and that seems to me a favorable precedent for it to be regarded as natural. The three kingdoms form three major series which begin with the least perfect beings and end with the most perfect. Under the similarity of their organizations, they are themselves made up of several series or smaller families which are united by beings which, although appearing to take the nature of two or several families, properly belong neither to one nor the other, and by this form transitions; this makes it difficult to find striking characters. Zoophytes unite the three kingdoms; mammals are united to fish by the whales, and birds to quadrupeds by bats, etc." *(Augier [[55], p.vii] as translated by Stevens [[43], p.206])

**Figure 5:**
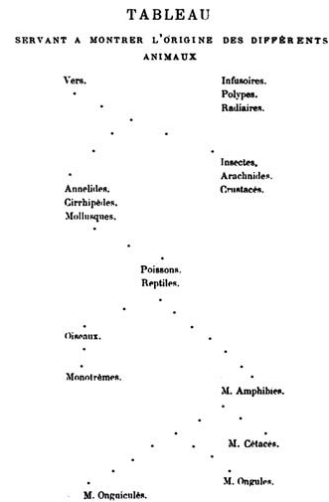
**Tree depicting the origin of animals, from the *Philosophie zoologique *of Jean-Baptiste Lamarck (1809)**.

**Figure 6:**
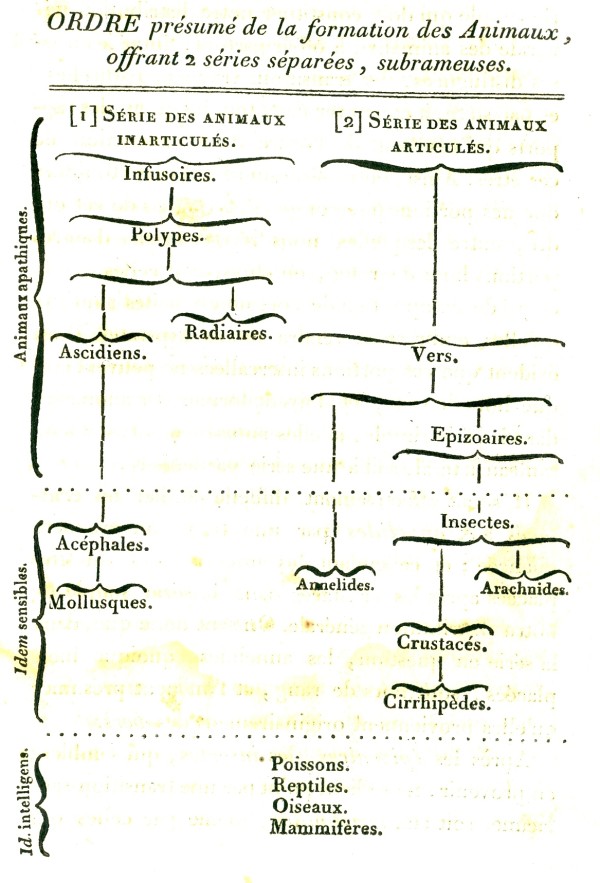
**More-detailed tree depicting two branching series of animal origins, from the *Histoire naturelle des animaux sans vertèbres *of Jean-Baptiste Lamarck (1815)**.

Stevens [43] finds no evidence that Augier intended this tree to depict an evolution or transformation of species. By contrast, Lamarck made evolutionary change, including the ongoing spontaneous creation of primitive forms and the upward transformation of existing life, the centrepiece of his evolutionary theory. As mentioned above, in *Flore Françoise *[14] he accepted a unitary scale of (plant) life. Later, in *Recherches sur l'organisation des corps vivants *[56] he drew back from fine-scaled continuity, although he continued to find continuity across major internal organ systems of animals [[57], p.128]. But to explain origins of specific groups of animals - cetaceans, for example - he found it necessary to invoke branches. Lamarck's first tree (Figure 5), presented in an addendum to his 1809 *Philosophie zoologique *[58], has few branches, whereas those in *Histoire naturelle des animaux sans vertèbres *[[59], I p.457] showed more (Figure 6). The titles of these trees reveal their evolutionary intent: *Tableau servant à montrer l'origine des différens animaux *and *Ordre présumé de la formation des Animaux, offrant 2 séries séparées, subrameuses *respectively. Lamarck's theory of evolution was widely known in his lifetime, but he persisted in linking it to idiosyncratic theories of chemistry and physics, and encountered scientific and political opposition from Cuvier and others [60,61].

Trees returned a couple of decades later as a depiction of the arrangement of nature (see below), but first we need to introduce the most important alternative: networks.

## Networks before Darwin

Remarkably, the network metaphor is older than that of a branching tree. In his 1750 *Della storia naturale marina dell'Adriatico *[62], Vitaliano Donati sought to describe the arrangement of aquatic organisms. He argued that regularities in nature allowed one to extrapolate from, or apply analogies based on, the terrestrial (hence more easily observable) Chain to understand the natural history of organisms in the sea, where it may be that transitions from the plant to the animal are most often encountered. But the result was not a single Chain encompassing all life, both terrestrial and aquatic:

*When I observe the productions of Nature, I do not see one single and simple progression, or chain of beings, but rather I find a great number of uniform, perpetual and constant progressions*. [[62], p.xx]

Moreover, these numerous progressions are interconnected:

*In each one of those orders, or Classes, nature forms its series and presents its almost imperceptible passages from link to link in its chains. In addition, the links of the chain are joined [uniti] in such a way within the links of another chain, that the natural progressions should have to be compared more to a net [rete] than to a chain, that net being, so to speak, woven with various threads which show, between them, changing communications, connections, and unions*. [[62], p.xxi]

Where in the second sentence above I translate *uniti *as *joined*, the 1758 French translation [63] renders it as *entrelacés*, interlaced or intertwined.

Giuseppe Olivi [[64], I p.68] likewise found the network an appealing metaphor for aquatic nature. Quoting Leibnitz to similar effect, Gottfried Treviranus wrote of nature as being made of "thousands and many thousands of chains, with endless skill entwined down to the smallest knot" [[65], I pp.473-475]. As this quotation is from the first book to use the word *biology *in a modern sense (*Biologie, oder Philosophie der lebenden Natur*), it can be said that nature-as-network was present at the dawn of modern biology.

**Figure 7:**
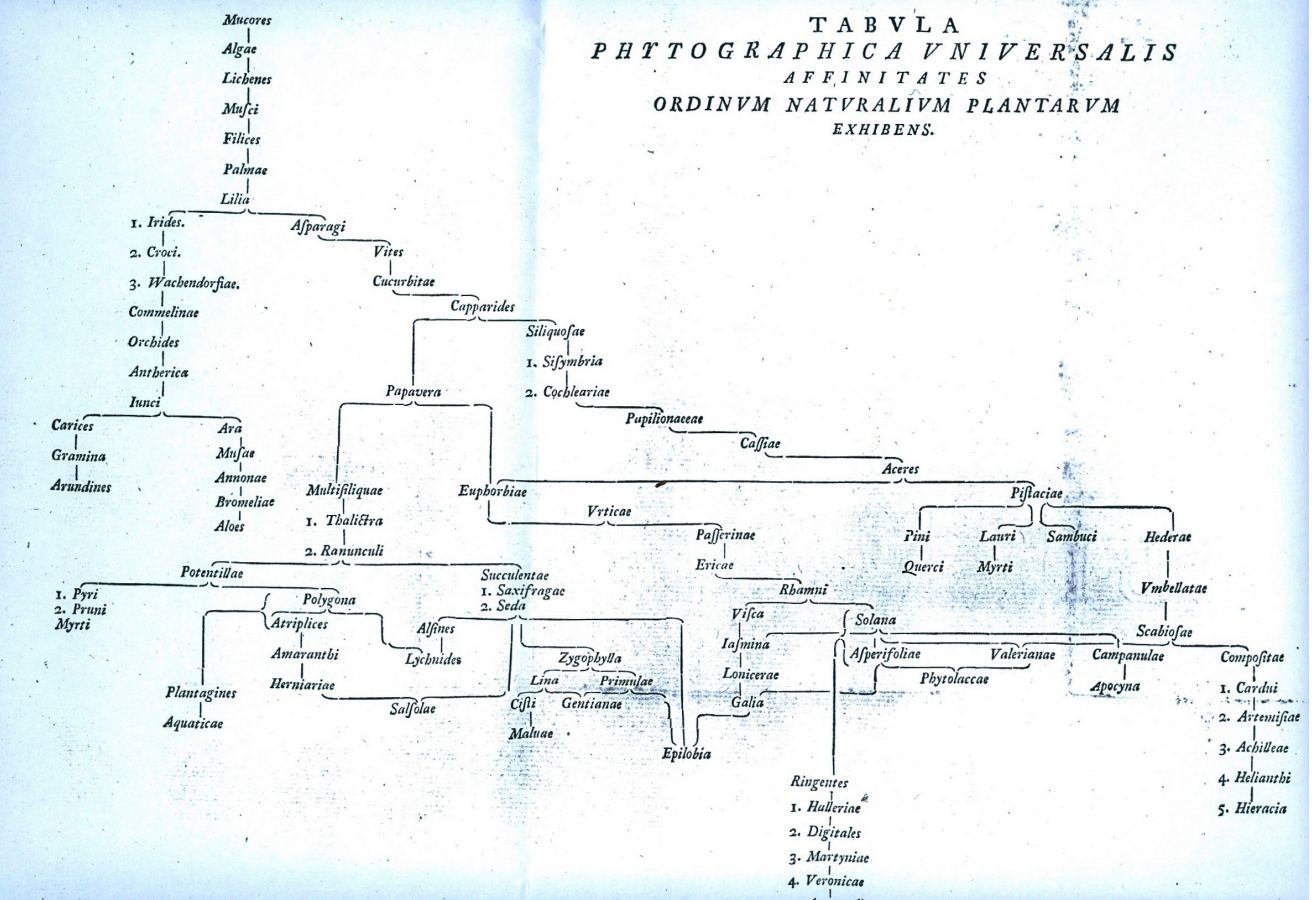
**Network of affinities among the natural orders of plants, from the *Ordines naturales plantarum commentatio botanica *of Johann Rühling (1774)**.

**Figure 8:**
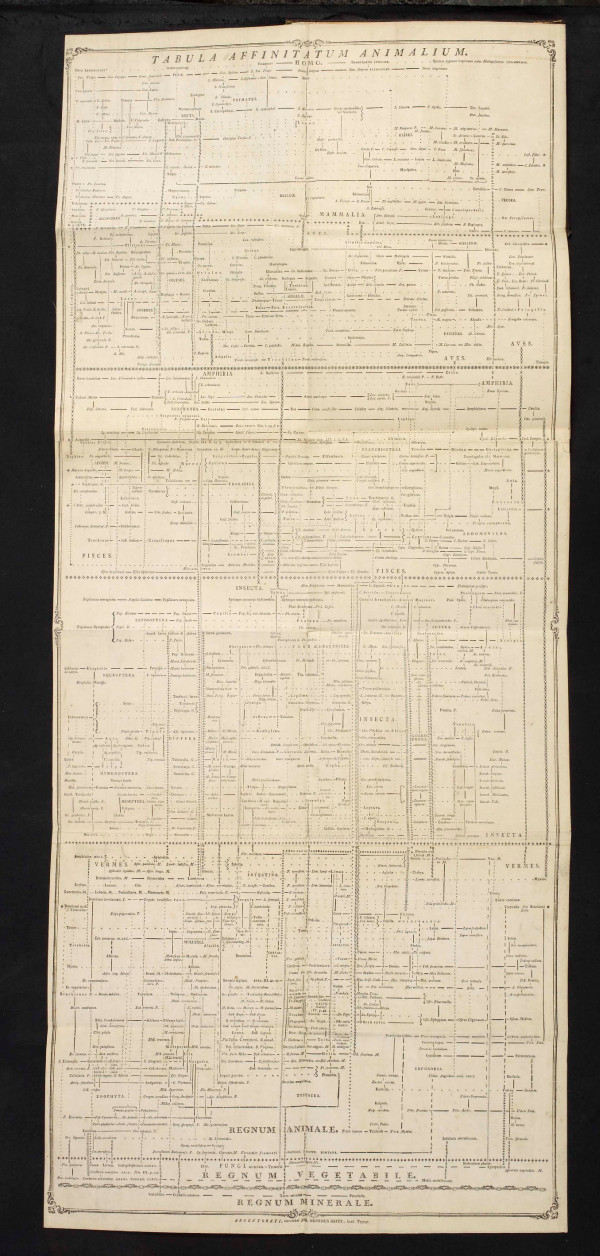
**Network of affinities among animals, from the *Tabula affinitatum animalium *of Johann (Jean) Hermann (1783)**.

**Figure 9:**
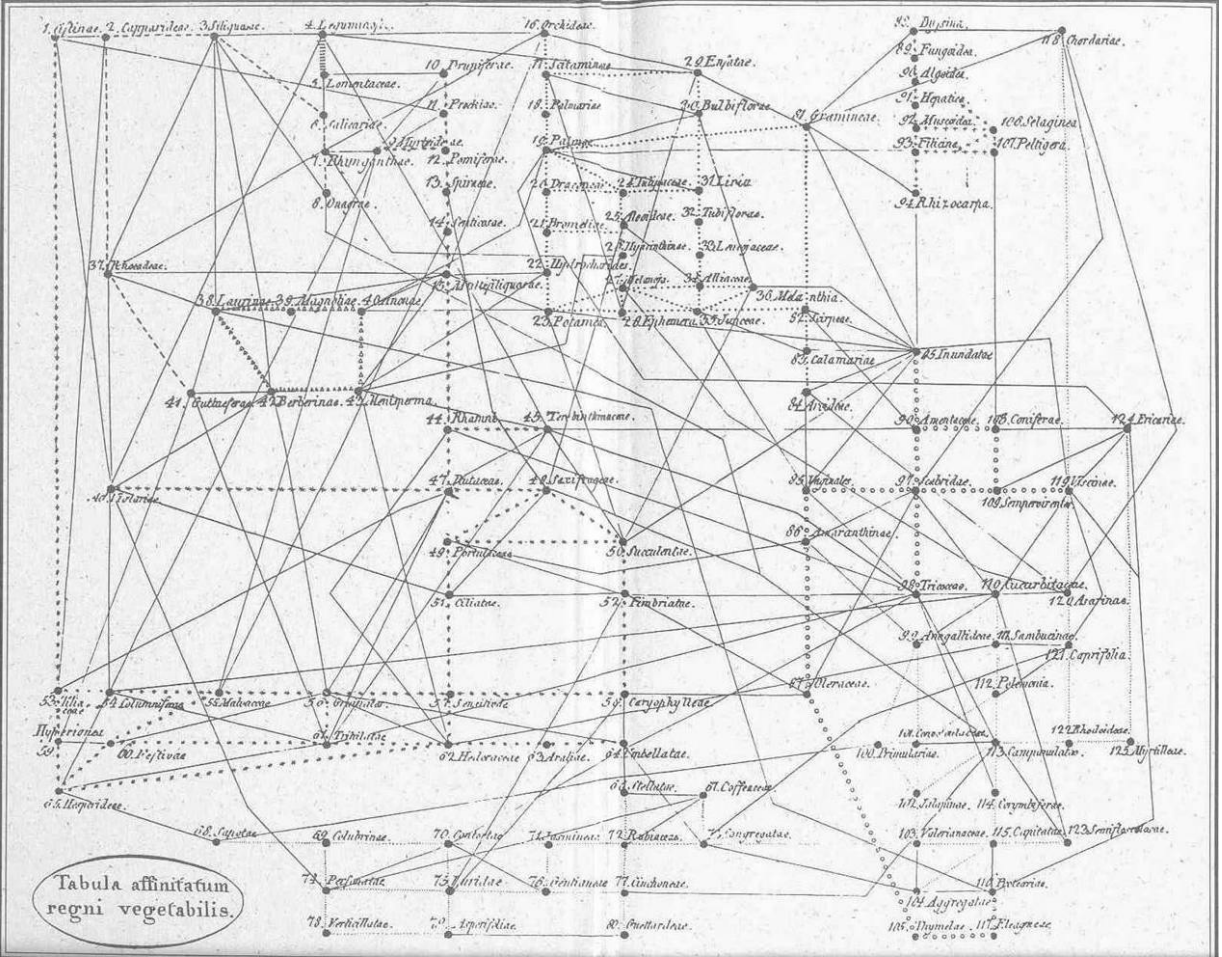
**Network of affinities within the vegetable kingdom, from the *Tabula affinitatum regni vegetabilis *of August Johann Georg Carl Batsch (1802)**.

We have already encountered the Eighteenth-century concept of *affinity *(above). Networks of affinities among plants and among animals were depicted in great detail in the late Eighteenth and very early Nineteenth centuries. Notable examples appeared in the *Ordines naturales plantarum commentatio botanica *of Johann Philipp Rühling (Figure 7) [66], the *Tabula affinitatum animalium *of Johann (Jean) Hermann (Figure 8) [67], and the *Tabula affinitatum regni vegetabilis *of August Johann Georg Carl Batsch (Figure 9) [68]. In the text Hermann identifies some of these affinities in detail, for example those which, in his opinion, link the rays (genus *Raja*) individually with Mammalia, Amphibia, Insecta, Aves, Pisces and Vermes [[67], p.295].

Treviranus argued [[65], I p.474) that whereas a chain allows one to describe only a single facet of organisation, with a network the complete organisation can be taken into account. He did not specify which organisational features might comprise the network of organisms, but the immediately previous section of his treatise discussed gradations in *e.g*. musculature, circulatory and nervous systems, and the brain. Nor did Treviranus propose that one organisational feature (say, musculature) could be associated with the *x*-axis of a network diagram, and a second feature (say, nervous-system organisation) with its *y*-axis; he meant instead that gradations in multiple - indeed all - features could be represented at fine scale, as adjacencies within local neighborhoods of the network. Nor, to my knowledge, did any network (or Linnaean map) proposed in these decades depict affinity on one axis, and analogy on the other.

Georges Cuvier later used the network analogy to emphasize the connectedness of all beings:

...*our systematic methods consider only the nearest affinities; they seek to place a being only between two others, and they are unceasingly at fault; the true method sees each being in the midst of all others; it shows all the radiations by which it is connected more or less closely within this immense network which constitutes organised nature *[[69], I p.569]

**Figure 10:**
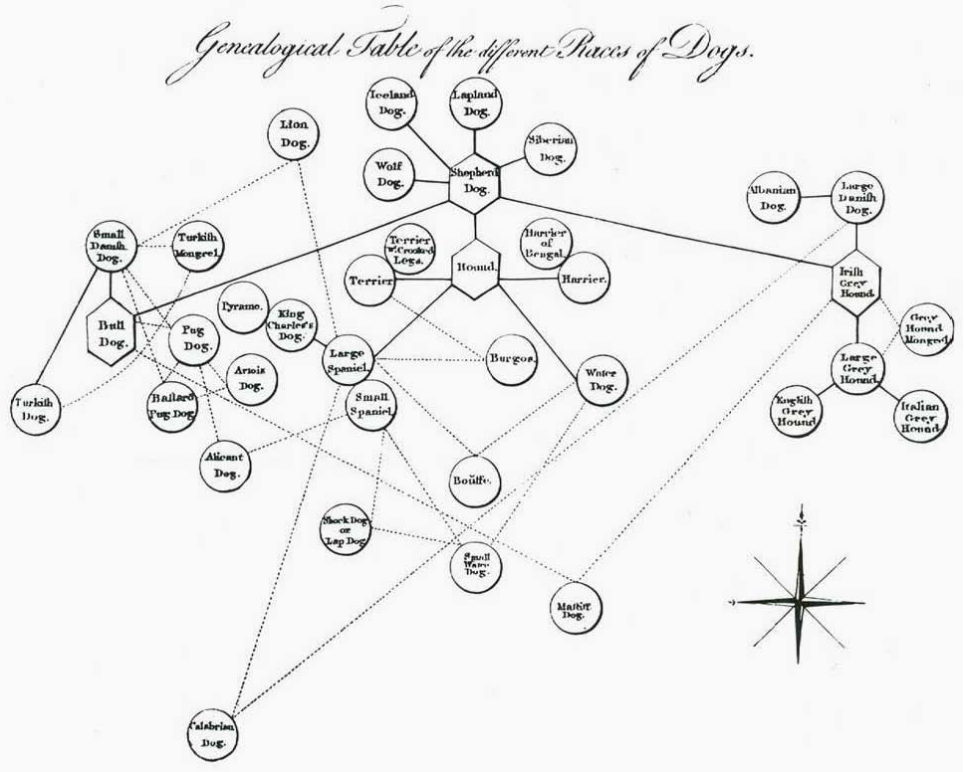
**Network of genealogical relationships among breeds of dogs, from William Smellie's translation of the *Histoire Naturelle *of Georges-Louis Leclerc, Comte de Buffon and Louis-Jean-Marie Daubenton (1753)**.

Networks appeared in a second, and very different, context shortly after 1750. Buffon held a somewhat idiosyncratic (and occasionally inconsistent) view of nature, believing in the fixity of species while refusing to accept the reality of higher taxa. Like Darwin much later, however, Buffon was interested in animal breeding and in the diversity of forms it could yield. Buffon's well-known diagram of relationships among breeds of dogs (Figure 10) [70] is not only a network but an explicitly genealogical one, albeit in Buffon's view a special case due to the human intervention that is required.

## Other representations of nature before Darwin

**Figure 11:**
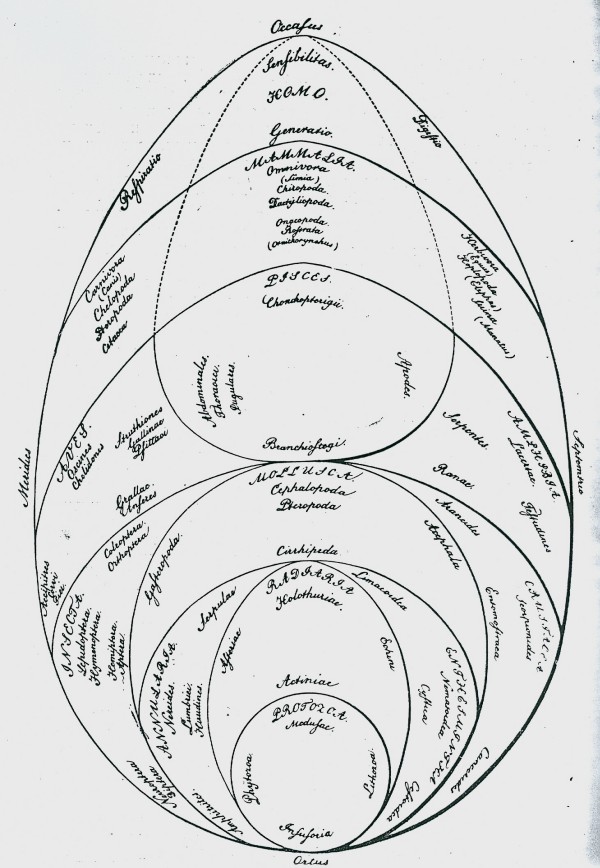
**System of animals, from *Ueber die Entwicklungsstufen des Thieres *by Georg August Goldfuss (1817)**.

**Figure 12:**
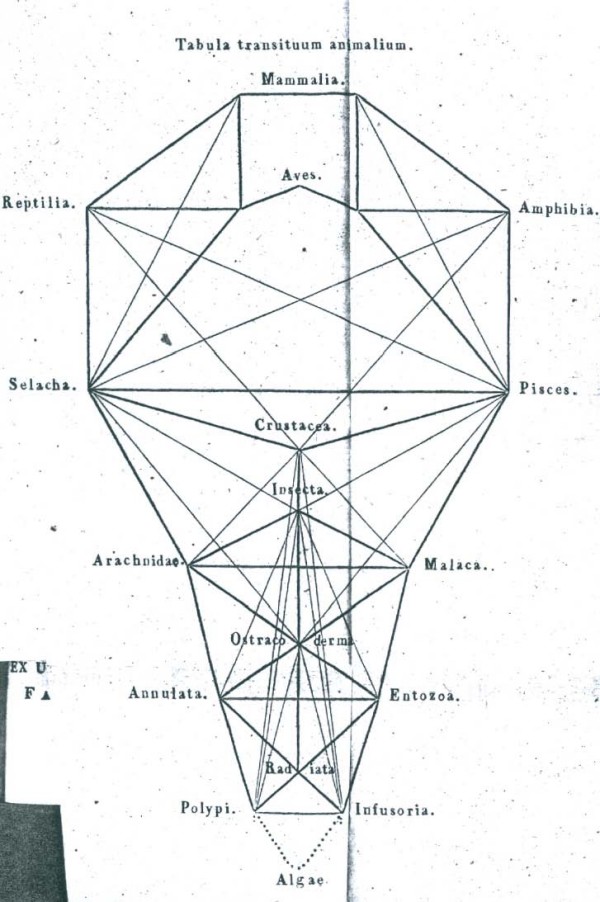
**System of animal "transits", from *De regni animalis limitibus atque evolutionis gradibus *by [Carl] Edward [von] Eichwald (1821)**.

**Figure 13:**
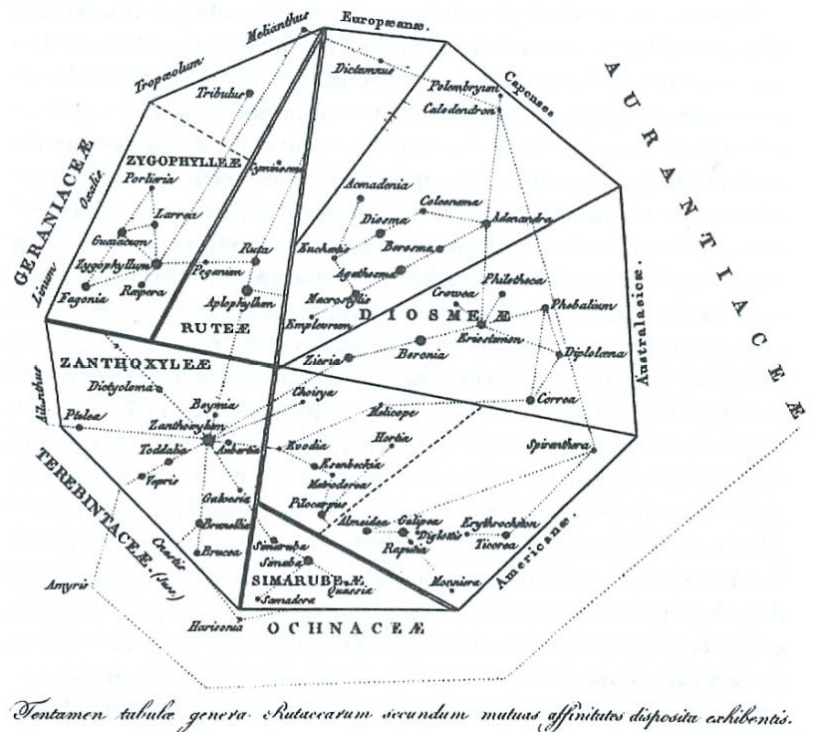
**Attempt at an account of the genera of Rutaceae shown arranged according to their mutual affinities, from Adrien de Jussieu (1825)**.

**Figure 14:**
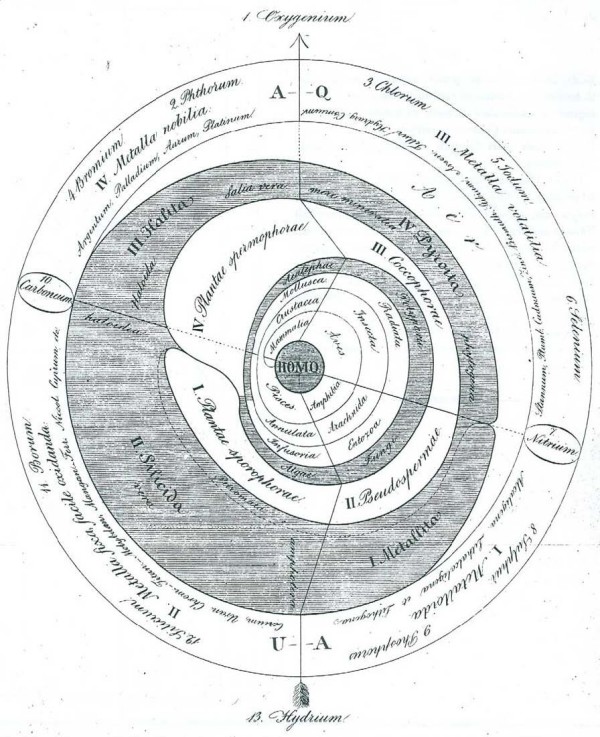
**Universal system of nature, from the *Primae lineae systematis naturae *of Paulus Horaninow [Pavel Feodorovich Ghoryaninov] (1834)**.

In the passage translated above, Pallas [48] refers to polyhedric representations of order in nature other than series, trees and networks. Here (as elsewhere) the 1787 German translation takes some liberty with the Latin text, elaborating that these polyhedric figures have multiple surfaces and compartments [[49], p.47]. I am unaware of pre-1766 representations that could have been so described, apart perhaps from a particularly angular conceptualisation of the Linnaean map. Fifty years later, however, we do encounter a diversity of polyhedric representations of the living world [54,71-73] (Figures 11, 12, 13, 14).

**Figure 15:**
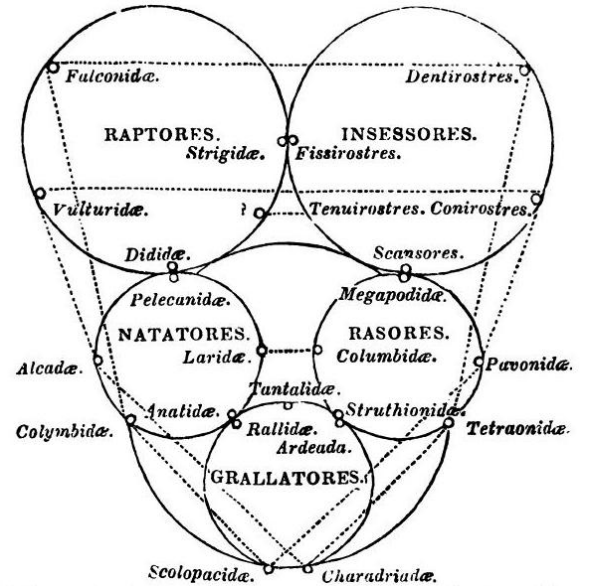
**Quinarian system of birds, from Volume 2 of William John Swainson's *On the natural history and classification of birds *(1837)**.

**Figure 16:**
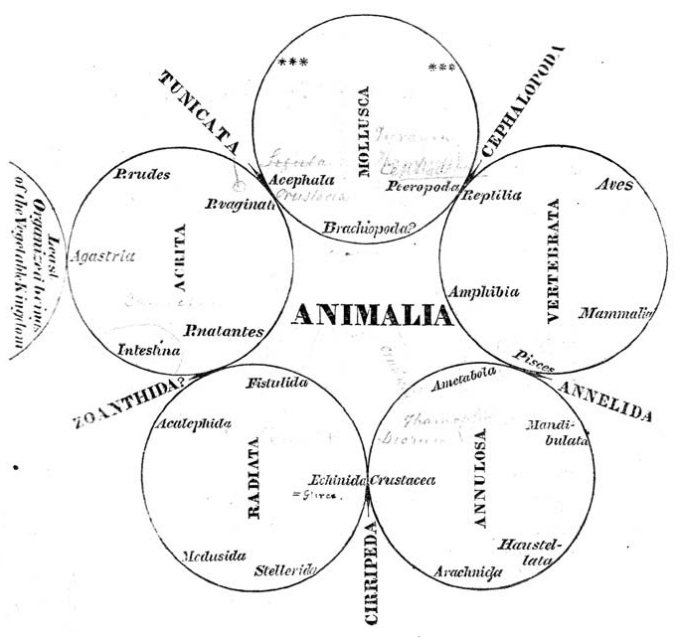
**Quinarian system of animals, from Volume I, Part 2 of the *Horae entomologicae *of William Sharp Macleay (1821)**. Handwritten annotations (not readily visible at this brightness setting) on this original, from the Fisher Library at the University of Sydney, may have been made by WS Macleay himself.

**Figure 17:**
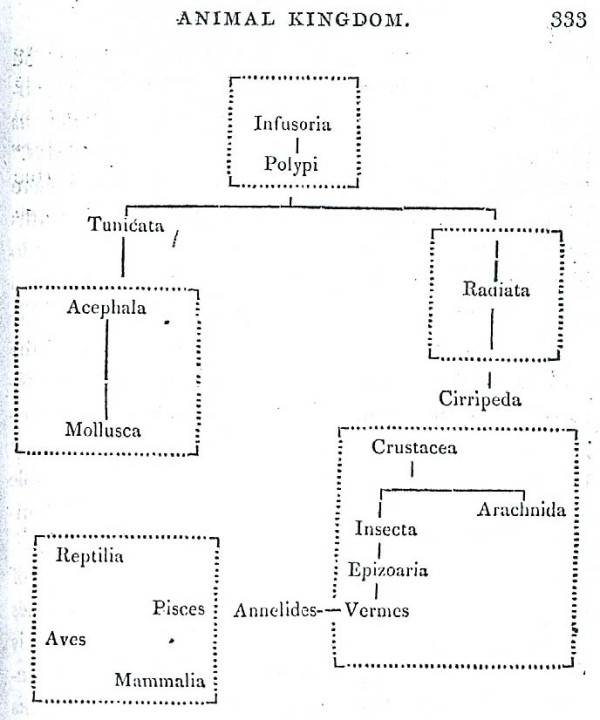
**Quinarian system of animals, from the *Horae entomologicae *of William Sharpe Macleay (1821)**. Macleay has superimposed his system on the 1809 evolutionary tree of animals of Jean-Baptiste Lamarck (*cf *Figure 5).

Notable among these alternative was the quinarian system, in which groups were arranged as a cycle of cycles. Swainson [[74], p.91] credits the modern idea to Fischer de Waldheim [[75], p.181], who in 1805 arrayed animals in "a series of contiguous circles around Man as a centre". As developed by Macleay [76], the animals are constituted as five great natural groups (classes Acrita, Radiata, Annulosa, Vertebrata and Mollusca), each of which is further divisible into five lesser groups; even the imperfectly known Mollusca were unlikely to constitute an exception to this "rule" [[76], p.321]. Arranging the five sub-groups in each class into a circle would reveal a series of affinities within that class; and arranging the five class-circles into a larger ring would reveal affinities between sub-groups in different classes, while identifying other similarities as mere analogies [[76], p.319]. These analogies moreover might be positioned non-randomly, *e.g*. forming axes as in Swainson [[77], p.200] (Figure 15). Certain otherwise problematic groups, such as Tunicata, could be placed as "osculant" groups at the tangent between adjacent classes. As in the Linnaean map, local affinities did not align head-to-tail into a unitary series or chain: note the order of classes Acrita → Mollusca → Vertebrata → Annulosa → Radiata (→ Acrita) in Macleay's system, with the vertebrate sub-groups Aves and Mammalia as distant as possible from the acritan sub-group Agastria, the point of contact with Vegetabilia [[76], p.318] (Figure 16). This allowed Macleay to argue that Lamarck's 1809 tree was really an un-recognised quinarian cycle (Figure 17); perhaps because he considered these regularities to be evidence of a divine design in nature [[76], pp.324-325 and elsewhere], he did not ask whether, conversely, his cycles might represent planar sections through Lamarck's trees. Similarly a Venn-like diagram of animal taxa based on embryology [78] might have been (but was not) presented as a planar section through a tree (or a sparse network, if its additional correspondences are to be included).

**Figure 18:**
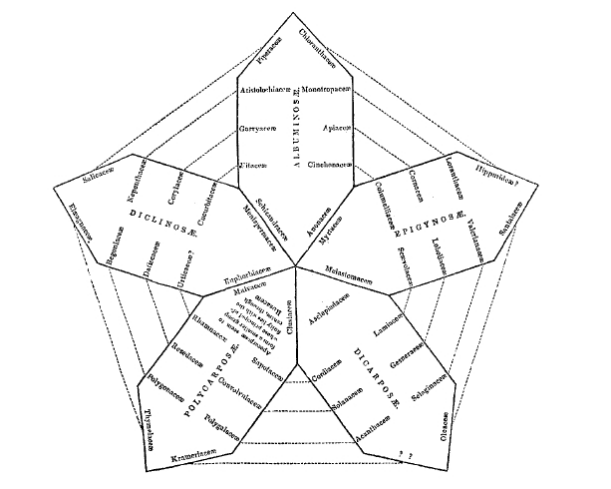
**Hybrid quinarian system of "exogens" (dicotyledenous plants) by John Lindley, from Volume 10 of the *Penny Cyclopædia *(1838)**. For detailed discussion see Coggin [81].

The quinarian system was adopted by Vigors [79], Swainson [77], Lindley [[80], p.130; [81]] (Figure 18), Kaup [82] and others, a seven-cycled system by Newman [83], and a ten-cycled system by Elias Fries [84]; however, following criticism by Strickland [85] in 1841 these systems began a terminal slide into disfavour. The quinarian system was nonetheless privileged with a lengthy chapter in the (in)famous *Vestiges *[86], and found its way into the popular literature in works such as Hugh Miller's *Testimony of the Rocks *[[87], p.493]. Darwin was thoroughly aware of the quinarian system, and worried that his own theory of natural selection might suffer a similar fate if presented carelessly to the public [[88], note 5]. The young Edward Drinker Cope sketched a quinarian classification of amphibia in 1857 [[89], p.258], and a variant the theory was taught at the University of Toronto as late as 1870 [81].

## Trees 1837-1859

O'Hara [[44], p.256] characterizes the years 1840-1859 as a "mapmaking" period, in which Strickland and Wallace sought to banish analogy and symmetry (hence quinarian circles and other geometric representations) from systematics. During these two decades at least four investigators published tree diagrams, two depicting stratigraphic series of fossils. From 1840 through at least 1856, in some thirty editions of his popular textbook *Elementary Geology*, Edward Hitchcock [90] presented a branching diagram representing series of plant and animal fossils in successive strata. These diagrams had been removed by the 1860 edition [91]. Archibald [91] notes that Hitchcock did not accept the transmutation of species, arguing against Lamarck, Chambers (*Vestiges*) and later Darwin. Louis Agassiz presented a similar tree-like depiction of fossil fish in the first volume of his *Recherches sur les poissons fossiles *[[92], pp.170-171]; like Hitchcock, Agassiz did not accept a transmutation of species.

**Figure 19:**
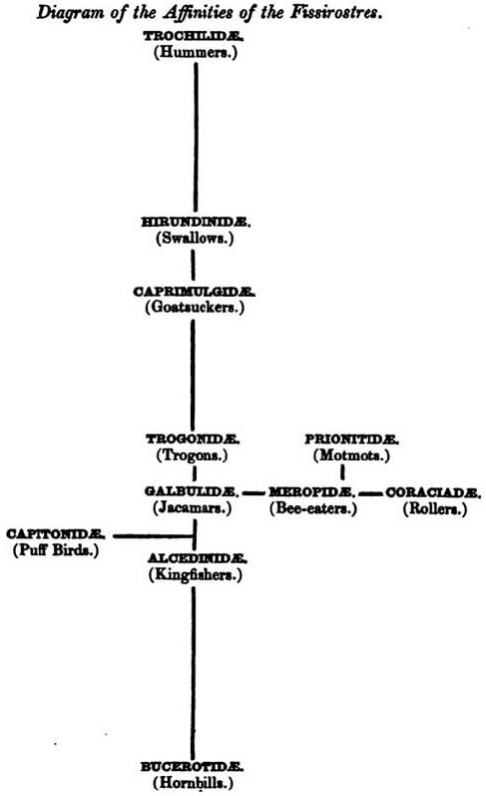
**Tree diagram of affinities of the bird group Fissirostres, by Alfred Russel Wallace (1856)**.

Alfred Russel Wallace, on the other hand, sought evidence for transmutation of species during explorations in Brazil (1848-1852) and the Malay Archipelago (1854-1862). In 1855 he communicated a paper referring to "branching of the lines of affinity, as intricate as the twigs of a gnarled oak or the vascular system of the human body", and later to "the true Natural System of classification" needing to order "the stem and main branches represented by extinct species" as well as extant diversity, "a vast mass of limbs and boughs and minute twigs and scattered leaves" [93]. In a remarkable paper published the following year, he offered not only branching representations of affinities among two groups of birds (Fissirostres and Scansores), but also an explicit description of his method of tree-building [[94], pp. 206-207]. Wallace accepted that there exists a main axis along which many taxa can be arranged in a series of affinities, while affinities of other taxa can be represented as branches and sub-branches to the left and right off this main axis. These affinities [[94], p.199] were of structure and "economy" [[94], p.195], the latter term connoting features of reproduction, nutrition, physiology and ecology; branch lengths correspond with degree of affinity, and gaps may occur at any point due to extinction of families [[94], pp.206,214]. As a consequence, Wallace's trees (Figure 19) were both phylogenies (in the modern sense of showing extant taxa at the branch-tips) and morphoclines. Wallace explicitly stated that his approach followed Lindley and Strickland against the quinarian system of Vigors and Swainson [[94], pp.195,206,212].

**Figure 20:**
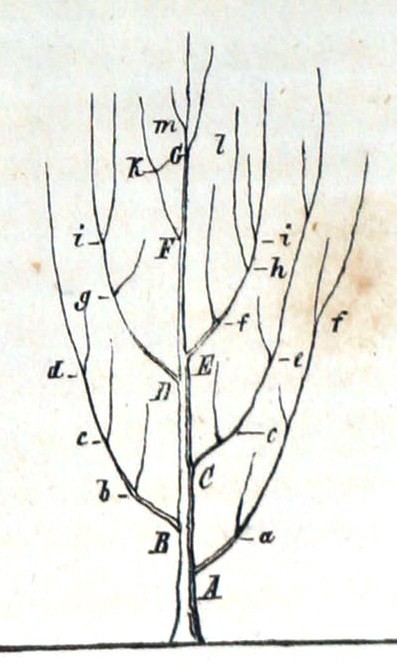
**System of animals, shown in the form of a tree, as evidenced by the fossil record; by Heinrich Georg Bronn (1858)**.

In 1858 Heinrich Georg Bronn [95] depicted a system of animals, as evidenced by the fossil record, in the form of a branching diagram drawn to resemble an actual tree (Figure 20) and described as a *Baum-förmige Bilde des Systems *with *Äste *(branches). His tree depicted a series of species, of increasing organisational perfection, succeeding each other as time progresses; this succession continues on all branches, although at any point in time some new species are necessarily more-perfect than others on lower branches [[96], pp.110-116]. Mikulinskii [53], p.316] notes that Bronn had earlier cited Eichwald's *Zoologia specialis *[51], hence presumably had studied Eichwald's (Pallas's) tree. Bronn later made the first translations of *Origin of Species *into German, adding footnotes and a chapter of his own criticisms; one of these translations was read by Haeckel [97].

## Darwin's dangerous trees

In 1837, in his *Notebook B *[[98], B§21], Darwin famously wrote

organised beings represent a tree. Irregularly branched some branches far more branched. - Hence Genera. - «as many terminal buds dying, as new ones generated»

**Figure 21:**
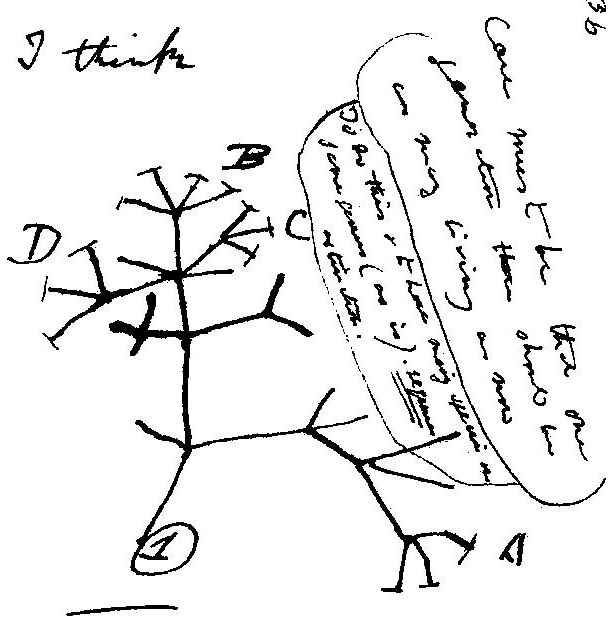
**The "I think" tree from *Notebook B *by Charles Robert Darwin (1837)**. Figure from Wikimedia Commons.

In successive notes (*e.g*. [[98], B§23,24]) he refers to "the tree of life", and wondered whether "(t)he tree of life should perhaps be called the coral of life" [[98], B§25]. Bredekamp [99] argues that the two small branching figures Darwin sketched first in Notebook B [[98], B§26] were in fact inspired by specimens he himself had collected and thought to be corals (some later turned out to be calcareous algae); on the other hand, Voss [100] links these sketches to embryological diagrams by the physician Martin Berry. Darwin's more-famous "I think" figure [[98], B§36] (Figure 21), while topologically a tree, does not resemble any actual botanical tree, nor does Darwin call it a tree. The mathematical structure today known as a *tree *(although not the term itself) was introduced a decade later by Kirchhoff [101] and von Staudt [[102], pp.20-21], while Arthur Cayley [103] is thought to have been the first to call this structure a *tree*.

**Figure 22:**
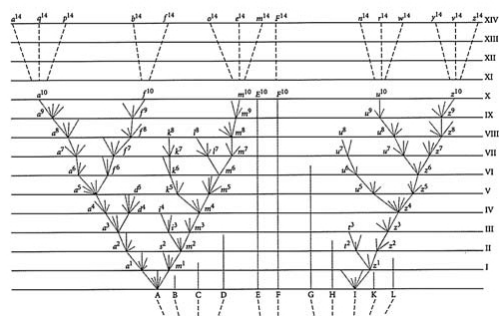
**Great Tree of Life, from *Origin of Species *by Charles Robert Darwin (1859)**.

The only illustration in *Origin of Species *was an abstract tree diagram (Figure 22):

*The affinities of all the beings of the same class have sometimes been represented by a great tree. I believe this simile largely speaks the truth. The green and budding twigs may represent existing species; and those produced during each former year may represent the long succession of extinct species. At each period of growth all the growing twigs have tried to branch out on all sides, and to overtop and kill the surrounding twigs and branches, in the same manner as species and groups of species have tried to overmaster other species in the great battle for life. The limbs divided into great branches, and these into lesser and lesser branches, were themselves once, when the tree was small, budding twigs; and this connexion of the former and present buds by ramifying branches may well represent the classification of all extinct and living species in groups subordinate to groups. Of the many twigs which flourished when the tree was a mere bush, only two or three, now grown into great branches, yet survive and bear all the other branches; so with the species which lived during long-past geological periods, very few now have living and modified descendants. ... As buds give rise by growth to fresh buds, and these, if vigorous, branch out and overtop on all sides many a feebler branch, so by generation I believe it has been with the great Tree of Life, which fills with its dead and broken branches the crust of the earth, and covers the surface with its ever branching and beautiful ramifications *[104,129,130].

Darwin's theory of evolution shared points of similarity with certain of the systems described in previous sections of this paper, but was unique in their combination. Like Lamarck and Wallace, he accepted the transmutation of species. Unlike Bonnet, Lamarck, Eichwald, Bronn and Haeckel, he rejected as a "miserable limited view" [[98], B§216] the ongoing creation or appearance of new species, instead emphasising the continuity of genealogical lineages from one or a small number of original forms. Darwin did not base his tree on affinity, although successive generations "tend to inherit those advantages which made their common parent (A) more numerous than most of the other inhabitants of the same country" [[104], p.118] (in this way, as Darwin put it, affinity will "grow" [[98], C§151]). His tree is instead one of genealogical inheritance. He placed extant taxa at the tips of his tree, not at the internal nodes or along the branches. Darwin described the struggle for existence in a Malthusian context, and importantly emphasised the central role of natural selection - his "dangerous idea" [[105], p.21] - without recourse to a metaphysical formative force toward perfection or realisation of potential *per *Bonnet, Lamarck, Eichwald and others.

## Trees and networks after Darwin

**Figure 23:**
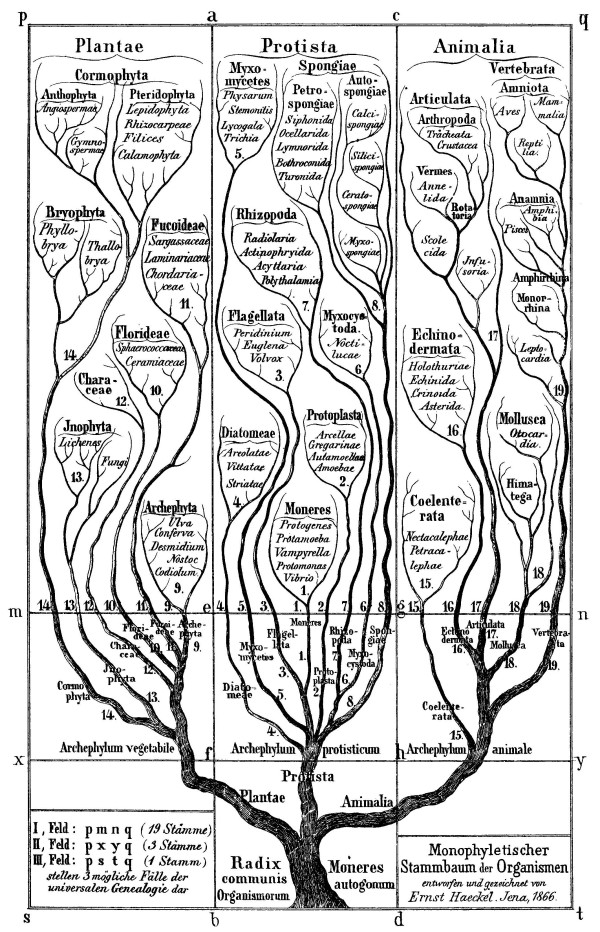
**Genealogical tree depicting three kingdoms of life, from Volume II of *Generelle Morphologie *by Ernst Heinrich Haeckel (1866)**.

Darwin attracted strong champions, including Thomas Henry Huxley in the English-speaking world and Ernst Haeckel on the Continent. Haeckel (following Bronn) did not accept every detail of Darwin's argument, but agreed that the natural system is necessarily genealogical, and seized enthusiastically on Darwin's abstract branching diagram as its form. Darwin had argued [104] that the geological record was too incomplete to provide a basis for fleshing-out this tree, so to speak, with actual taxa. Haeckel would instead base his reconstruction method on his so-called biogenetic law: the claim that an individual's ontogeny recapitulated, or re-traced and summarized, its phylogenetic history [106]. In this way he derived *Stammbäume *(genealogical trees), which he depicted as a proper botanical trees [106,107] to which extant biological taxa were assigned (Figure 23). His initial trees were refined and extended in great detail to numerous taxa. Note that Haeckel did not call his trees *phylogenies*, reserving this term for a series of morphological stages [108]; how could ontogeny (a linear process in a single organism) recapitulate phylogeny, unless phylogeny too was linear? The received meaning of *phylogeny *has since evolved, of course, and is now used to mean *genealogical tree*, even in contexts where only the branching topology is relevant.

Although it lies outside the scope of this article to review in detail the uptake of Darwin's theories and adoption of the evolutionary tree as an explanation of modern biodiversity, I offer three case studies to illustrate that this uptake and adoption progressed at different rates and to varying extents, if at all, across different fields of biology. Network diagrams [109] and geographical maps persisted in some research communities; and today, as we shall see, network representations are again resurgent in microbial phylogenetics [110].

### Vertebrates: trees in the ascendancy

Haeckel's visually striking and highly detailed trees of vertebrates (*e.g*. [106,107]) were widely discussed, not least because they ventured to show the genealogical history of man. By the later Nineteenth century it was reasonably commonplace for monographs on vertebrate zoology to include a branching tree of taxa. Debate persisted on important concepts: Garrod [111], for instance, argued that these trees must be interpreted as showing relationships among characters, not necessarily among taxa. Sharpe [112] depicted Reichenow's *Stammbaum *of class Aves as small boughs emerging at multiple points from four thick, poorly differentiated basal "stems". In the same work he also [112] represented a gracile, three-dimensional tree of birds as views from the front, back, side and top, adding planar sections at three different levels that linked the underlying cladogenic process to map-representations. Some of these figures have been reproduced and discussed by O'Hara [44,113]. Longitudinal sections were explored in more detail in the early Twentieth century, for example to illustrate polyphyly and convergence [114]. Other dynamics continued to play out, *e.g*. between topology-centric interpretation emphasizing speciation (cladogenesis) and anagenic change along branches (phylogenesis *in sensu *Haeckel). Trees were sometimes annotated to show character distributions, geographical spread, or quality of evidence [114]. Nonetheless the genealogical tree soon became established in vertebrate zoology, as indeed in many other biological domains.

### Algae: morphoclines and networks

Unlike vertebrates, algae are not a monophyletic group. Linnaeus [115] followed earlier authors in collecting various macroscopic "least-perfect plants" into form-genera, the number of which was revised upward or downward as his system was refined [37]. With the dissolution of the Great Chain, these form-genera were dissolved, and the new groupings that replaced them were augmented with *monads *(unicellular forms), a term that "thanks to Leibniz [116] and the *Naturphilosophes*, bore strong connotations of primitivity and potential" [37]. Some of these new taxa began to be viewed as "the lowest step of the plant kingdom", *i.e*. as archetypes for the land plants [[117], p.1]. And plants they were agreed to be: Haeckel [106,118] excluded brown and red algae, and macroscopic green algae, from his new kingdom Protista. Lamouroux [119] had introduced colour of spores and thalli as the key taxonomic feature, therewith recognizing Fucacées, Floridées and Ulvacées; this approach had practical value (*e.g*. identification in the field) and immediately suggested how unicells should be classified: yellow-brown unicells with Fucacées, red with Floridées, and green with Ulvacées.

**Figure 24:**
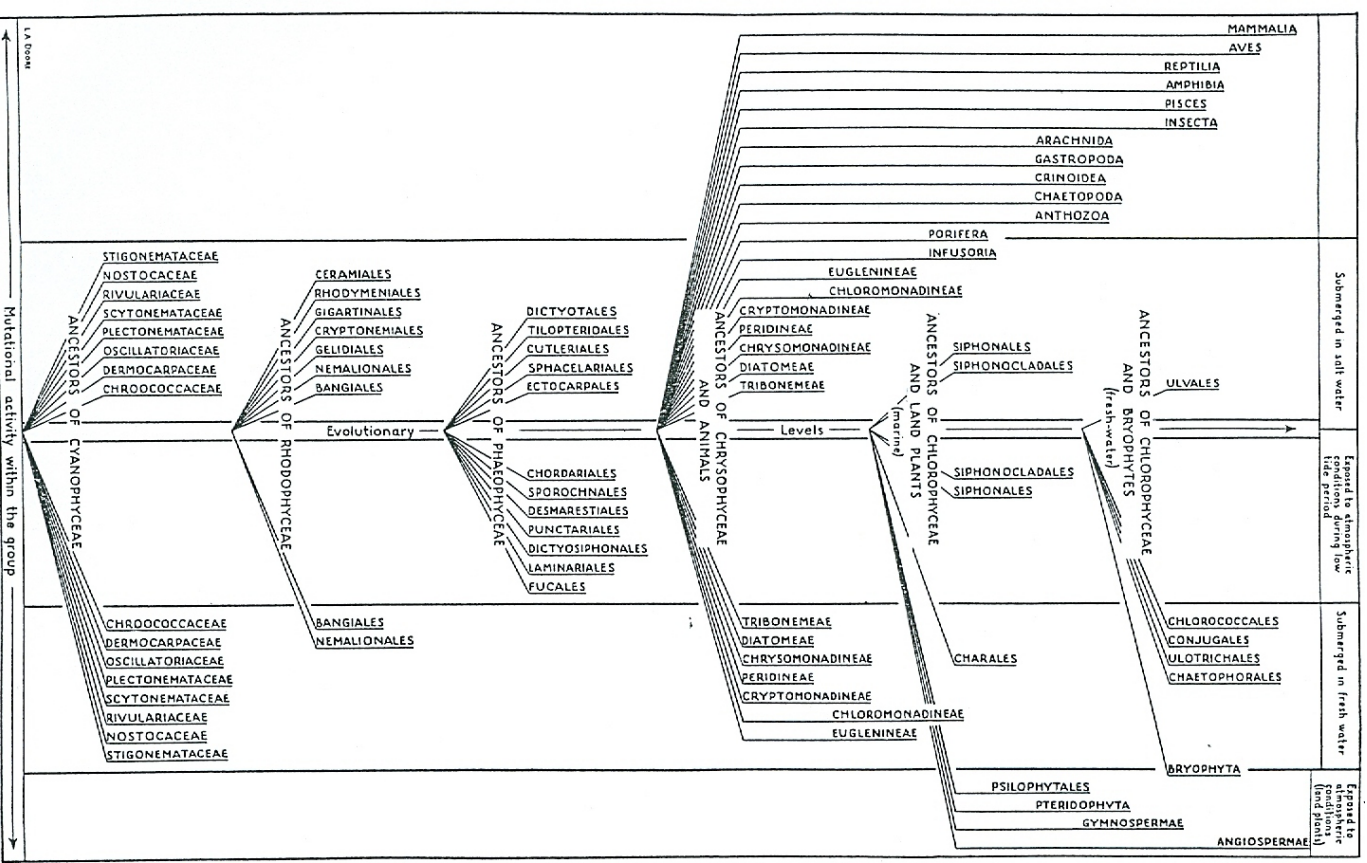
**"A diagram showing the evolutionary levels of the various algal groups and the mutational activity within each group" from *The Algae and their Life Relations. Fundamentals of Phycology *by Josephine E. Tilden (University of Minnesota Press, 1937)."** The most recently evolved members of the group (Chroococcaceae, Bangiales, Ectocarpales, etc.), shown nearest the center line, are the simplest in form and structure and represent most closely the ancestral type in each case. The oldest or earliest evolved members of the group (Stigonemataceae, Ceramiales, Fucales, etc.), shown farthest from the center, are the most specialized, having undergone, through mutational variations, a more or less complete change from the ancestral type." (Tilden 1937:5). The figure shown is Plate II, page 40; used by permission of the University of Minnesota Press.

**Figure 25:**
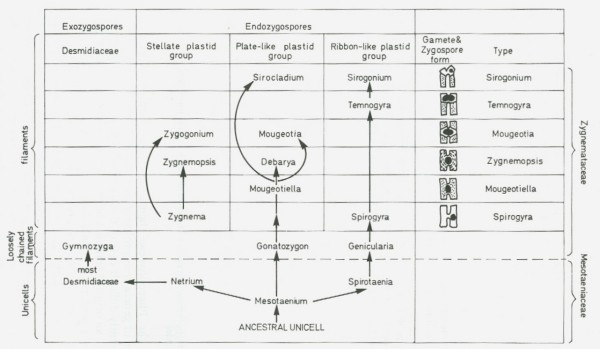
**Evolution of Conjugales, from Valentine J. Chapman and David J. Chapman, *The Algae *(1973:334)**. This figure is representative of evolutionary tree-diagrams in the phycological literature from the late 1800s through the late 1900s in depicting parallel morphoclines of (usually) increasing complexity along which extant species can give rise to others. Macmillan and Co. Ltd, reproduced with permission of Palgrave Macmillan.

Given their substantial diversity, morphological plasticity, complex and mysterious life histories, and near-absence from the fossil record, one can understand why algae were, and continued to be, arranged along morphoclines within each colour-group. In discussing green or red algae, for example, monographs and textbooks began (and still begin) with unicellular forms, followed by colonies of unicells, unbranched and branched filaments, sheets, and finally thalli of increasing differentiation [*e.g*. [120], pp.12-28; [121,122]] (Figures 24, 25). Even in the late Twentieth century, some authorities held extrinsic factors (competency, aesthetics, tradition, didactics, accepted paradigms, and history of practice) to be paramount ("taxonomy is an art and taxonomists are thus artists") [[123], p.428]. Many phycologists considered the phylogenetic perspective (in the sense of branching trees) an unattainable ideal, or indeed distrusted it.

**Figure 26:**
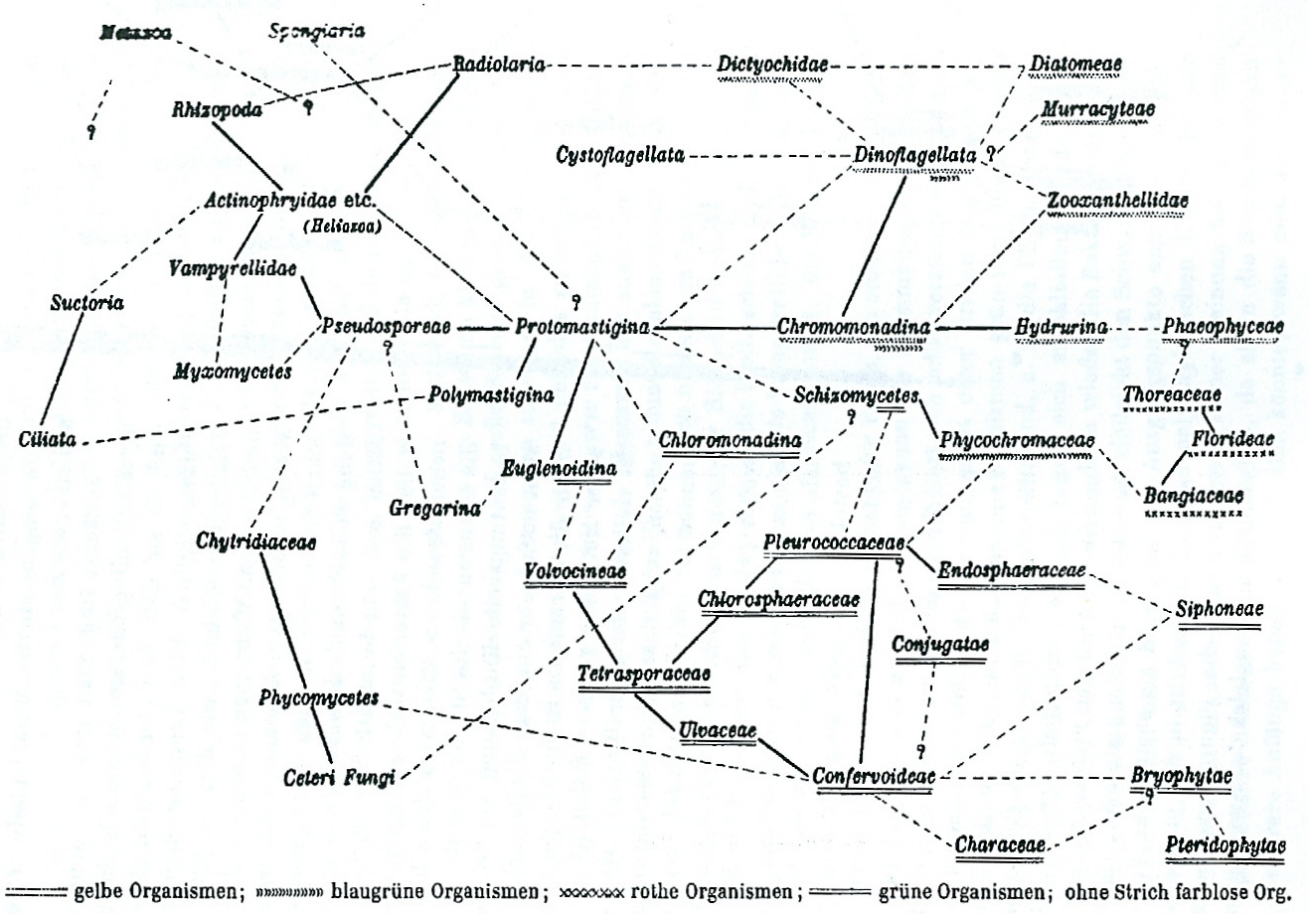
**Network of lines of relationships among groups of algae and protozoa, by Georg Klebs (1893)**.

Unicellular algae proved to be particularly problematic to bring into a phylogenetic system, with the already difficult biology complicated by a tradition - persisting to the present day - of including not only brown, red and green forms but also cyanobacteria and diverse protists (diatoms, xanthophytes, euglenoids) as "algae" in university courses, textbooks, societies and conferences. A number of these unicellular forms have colourless variants, thereby undermining the generality of pigmentation as a taxonomic character, while others (cryptomonads, chlorarachniophytes, some dinoflagellates) are the products of secondary or tertiary endosymbioses [124-126]. More than a century ago Klebs [127] depicted relationships among unicellular algae as a network (Figure 26), acknowledging nonetheless that it was "conceivable" that the "indubitable network of lines of relationship" among Pleurococcaceae, Volvocinae (and therewith the flagellates) and Confervoideae could be explained by *Transmutationslehre*, *i.e*. the theories of Darwin and Haeckel.

### Prokaryotes: disorder in nature

Microbiology developed largely after 1859 based on the work of Cohn, Pasteur, Koch and others. Few if any pre-1859 concepts were especially useful for systematizing organisms that could scarcely be seen under the microscope and presented few morphological characters. Further physiological characters were eventually identified, but they did not correlate in any simple or necessary way with what could be seen of bacterial morphology, and "every one of these characters is liable to variation" [[128], p.6]. By the mid-1920s it had become clear that at least some morphological and physiological characters of bacteria were rapidly and permanently transmutable under laboratory conditions [128-131]. The problem space was sharpened by excluding viruses, as non-living entities (but *cf*. [132]), from the microbiological system; but early expectations that a phylogenetic classification would emerge for bacteria [133] were unrewarded, and by the mid-Twentieth century there was considerable pessimism that a natural evolutionary classification of bacteria was even possible [134-139].

DNA was by this time known to be the genetic material, and DNA-DNA hybridisation experiments revealed, if crudely, a hierarchical structure of genetic similarity among bacteria [140-142]. More interestingly, given the structure of DNA, the nature of the genetic code and details of transcription and translation, protein sequences were "documents of evolutionary history" [143]; statistical approaches were developed to infer this history from sets of homologous sequences, and believable trees of relationships could often be inferred for eukaryotes, especially animals [144-149]. By contrast, however, the initial applications to prokaryotes were often less than successful due to restricted or sporadic distribution of genes, uncertain orthology, and/or lack of phylogenetic signal [150-153].

**Figure 27:**
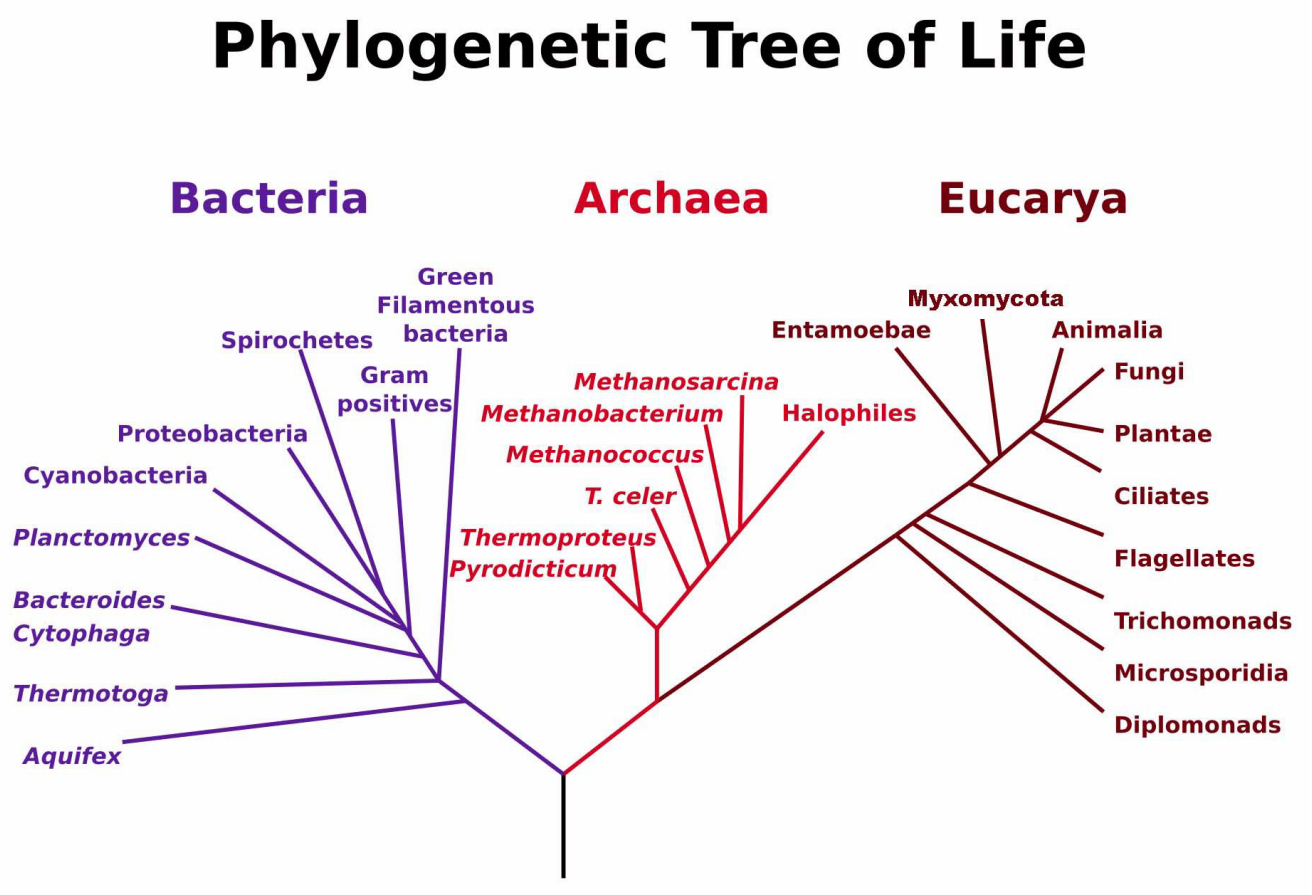
**Phylogenetic tree based on small-subunit ribosomal RNA sequences showing three domains of life**. Figure from Wikimedia Commons after Carl Woese and colleagues [186].

It was against this background that George Fox and Carl Woese initiated comparative analyses of RNA sequences [154-156]. In particular, the recognition of small-subunit ribosomal RNA (ssu-rRNA) as a universally distributed, well-behaved "molecular chronometer" [157] led to considerable optimism that the natural evolutionary classification of bacteria was finally at hand. This is what seemed, at first, to happen: Woese and colleagues delineated hierarchical groups among bacteria, identified the bacterial groups from among which chloroplasts and mitochondria had arisen (see also [158]) and, more controversially, reported that certain accepted groupings of bacteria (indeed "bacteria" themselves) were not, according to ssu-rRNA, monophyletic [157,159-161]. By the late 1980s the iconic "three kingdoms tree" of ssu-rRNA sequences (Figure 27) was increasingly assumed to be the Tree of Life. Indeed ssu-rRNA sequences began to serve as proxies for the corresponding organisms, allowing bacteria recalcitrant to isolation or culture, or unseen within a complex environmental sample, to be brought into the new phylogenetic framework [162].

**Figure 28:**
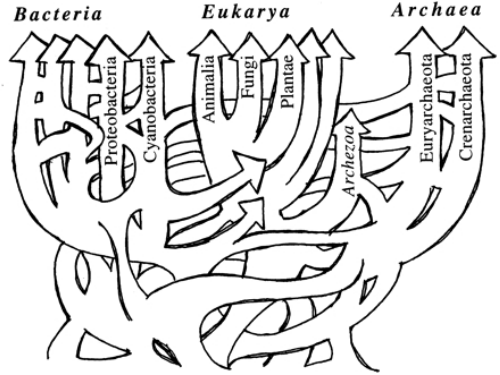
**"A reticulated tree, or net, which might more appropriately represent life's history", by W. Ford Doolittle**. Figure reproduced from [171] by permission of the American Association for the Advancement of Science.

**Figure 29:**
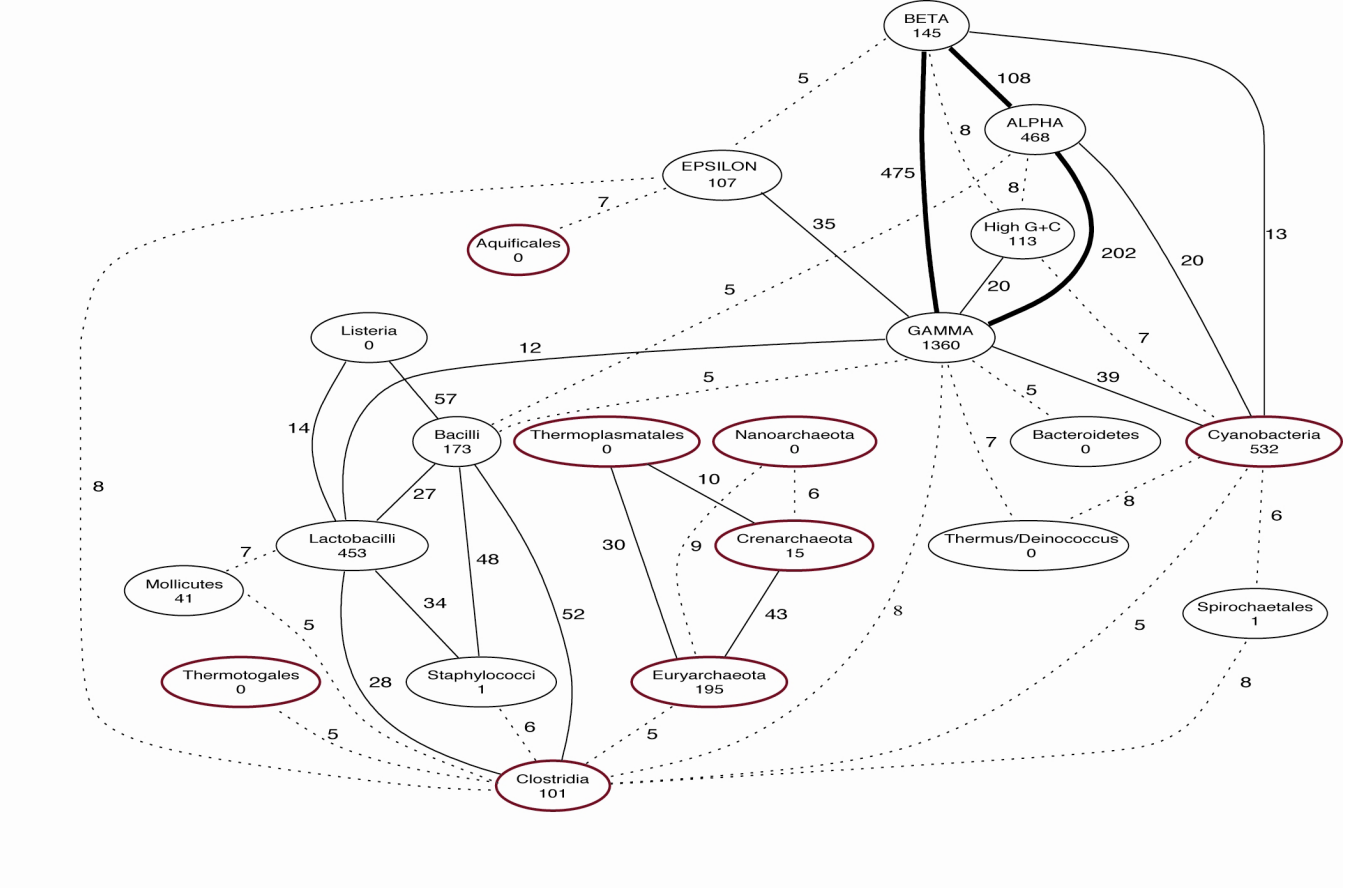
**Network showing major ("highways") of gene relationships in prokaryotes, by Robert Beiko**. Figure reproduced from [165].

**Figure 30:**
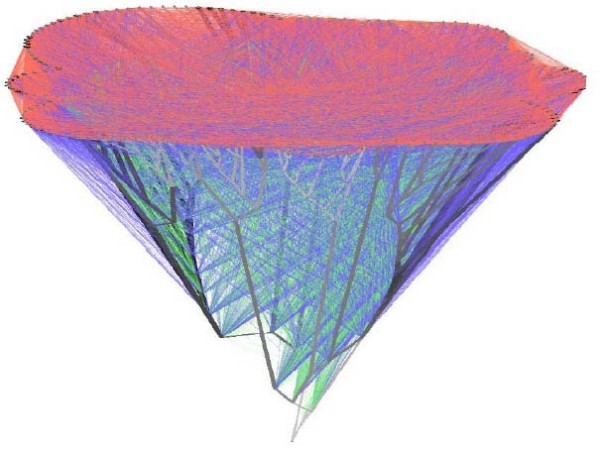
**Network representation of vertical inheritance and lateral exchange among prokaryotes, by Tal Dagan and William Martin**. Reproduced from Figure 2(e) of [167] by permission of the Royal Society.

As further sequences became available and phylogenetic inference grew more-sophisticated, trees were inferred for numerous gene families. Against almost universal expectation (but *cf*. [163]), however, these single-gene topologies failed to tell a fully consistent phylogenetic story, particularly for prokaryotes. Patterns of topological incongruence among different trees, or between a gene- or protein-family tree and a reference topology, came to be interpreted as *prima facie *evidence for lateral (horizontal) genetic transfer [164-171]. Genome histories that include both vertical (parent-to-offspring) and lateral components take the form of networks [110,165-172] (Figures 28, 29, 30). Scope remains for further methodological improvement, for example in better delineating the genomic units of phylogenetic analysis [173] and making better use of gene-content and gene-order data [174]. The final story may not have been written, but it is not unthinkable that we are witnessing the demise of the universal tree-of-life paradigm [171,175]. At minimum, the Tree of Life may "emerge, if at all, from the multi-genome era much more restricted in scope, and subject to many more qualifications, than could have been anticipated a dozen years ago" [[110], p.2169] - for example, as a Network of Life within which some localities and regions may, at some scales of resolution, be largely treelike.

## Conclusion

Darwin's trees played an integral role in his theory, linking process (genealogical descent-with-modification, plus extinction) with outcome (the diversity of past and present-day species, expressed as hierarchical systems of classification). Trees also provided a common, coherent, and (although this would be discovered only later) mathematically and statistically tractable framework linking much outside his argument *per se*, from comparative anatomy and development to palaeontology and biogeography, to evolutionary theory, with the result that now "nothing makes sense in biology except in the light of evolution, *sub specie evolutionis*" [[176], p.449]. Darwin's trees are justly considered a landmark not only of biology and of science more broadly, but of modern intellectual and visual culture as well [100,177].

In this article I have attempted to situate Darwin's trees in historical context, specifically the search for the natural system following the abandonment of the Great Chain of Being. His trees existed, and exist, in other contexts (*e.g*. sociological, political, religious) which I do not consider here. Darwin was not the first to propose that the system of nature is tree-like, nor that species undergo transmutation along hierarchically branching temporal trajectories. In the decades following 1859, genealogical trees won acceptance in some but certainly not all areas of biology; nor indeed have trees won full acceptance even today, although they remain default hypotheses for most biologists, as indeed more broadly in science and in society. But nature-as-network preceded the branching tree, was never completely supplanted by trees, and seems set to re-emerge as the most-inclusive metaphor for the living world - the "Network of Life" [110].

As we commemorate Darwin's birth and the *Origin of Species*, we also look forward to the 375^th ^anniversary, next year, of *Historia naturae, maxime peregrinae *and Nieremberg's hint at an unbroken web of nature.

## Reviewers' comments

### Reviewer's report 1

Dr Eric Bapteste, Dalhousie University, Halifax, Nova Scotia, Canada

Reviewer comments

This article offers a broad overview of the use of trees and networks in biology. The figures compiled by the authors are fascinating, and the demonstration that the network is not an odd concept for classifiers (and for evolutionists) is of significant importance. I encourage all curious biologists to read this piece.

1. What about the (endo)symbiotic theories and their support for networks after Darwin? Although their impact became significant sometimes only with delay, they are very briefly mentioned here. They have however certainly contributed to the debate around the tree-like *vs *web-like nature of evolution/classification (and for some of them, they have produced historically important drawings, such as Mereschkowsky's anastomosing scheme (and its multiple roots), or even such as Dagan and Martin webs of genomes, which recently made the cover of *Philosophical Transactions of the Royal Society of London B: Biological Sciences*. Thus would not it be worth, for instance, to discuss the contribution of Sagan/Margulis to the issue of network after Darwin a bit more? On p.2, the author suggests that "networks were reintroduced in the mid-1990", could not it be argued that with Margulis' piece and the subsequent studies promoted by her hypothesis, networks were reintroduced before? Finally, any contemporary web of genomes might be too recent to deserve a room in this historical paper, but I wonder whether including Dagan's web of genomes as a final picture of a biological network would not be appropriate.

Author's response

I am delighted to acknowledge the important work by Lynn Sagan (now Margulis) [178,179] which catalysed my own interest in the endosymbiotic theory of eukaryotic origins. Her argument encompassed the origin of eukaryotic cells by serial endosymbioses, the stepwise origin of mitosis, the non-existence of certain hypothetical intermediates, implications for high-level classification, and correlations with geochemical conditions on Earth - but was never, to my knowledge, explicitly abstracted as a story of network *versus *tree. Instead she mapped eukaryotic diversity as a unitary tree of mitosing cells, into which endosymbionts arrived at defined points on certain branches. I am likewise pleased to call attention to the visually compelling network diagrams by Dagan and Martin recently featured on the cover of *Philosophical Transactions of the Royal Society of London B: Biological Sciences *(volume 364 number 1527, 12 August 2009). Addressing the Reviewer's point more broadly, I have now added a ribosomal RNA-based "three kingdoms tree" (Figure 27) and three network diagrams (Figures 28, 29, 30) including one from Dagan and Martin (Figure 30).

Reviewer comments

I am a bit puzzled by the description of Aristotle's works only as supporting a great chain of beings to classify every taxon. I think this is too partial a view of his deep - and influential - philosophical analyses. In my understanding (and I believe consistently with what is found in the philosophy textbooks), Aristotle is known for having produced multiple classifications of beings, based on different characters. In short, Aristotle proposed multiple independent trees of beings, at a very high taxonomic level. Yet, he did not build them in an inductive way. It may be worth considering this latter point a bit more. That Aristotle was somehow a taxonomical pluralist (aka an author attracted by the reconstruction of multiple "nascent" trees) shows that (i) the question of adopting one *vs *many classifications, and (ii) the question whether these classifications were natural *versus *conventional/arbitrary systems, are also part of an ancient debate in biology, directly connected with the use of trees and networks in biology. I am thus curious to know, for each of the historical drawings presented in that manuscript, whether these schemes were considered by their authors as the natural picture of diversity, or mostly as some practical/"arbitrary" representations (of which they may even have proposed simultaneously many).

Author's response

Aristotle's Great Chain is not a classification: indeed in *De partibus animalium *(643b10-644a11) he argues that it is impossible to construct a single, logically consistent classification of organisms by dichotomous division, and that no series of *differentiae *can express the essence of an animal species. Some commentators have argued that there coexist in Aristotle both technical and non-technical senses of his terms *genos, eidos*, and *diafora *(genus, species, differentia) [[180]; [181], p.53]. Aristotle did present logical hierarchies for different overlapping sets of animals based on their sensitivity, attachment, motility, ovipary or other characters, and I agree that one might ask whether this makes Aristotle the first taxonomic pluralist. Of course none of this prevented various Sixteenth to Eighteenth-century authors from presenting unitary "Aristotelian" classifications of animals.

The question of whether these 30 figures, and indeed hundreds of others, depict their authors' view of the natural system, or instead were intended to serve only a limited utilitarian purpose, must remain beyond the scope of this paper. Herbals and bestiaries were utilitarian, as in general were keys such as the one from Zaluziansky (Figure 3); and Lamarck is said to have considered his early scale of plants [14] as "unscientific".

Reviewer comments

The author indicates that Lamarck's trees are a temporal progression. This is not entirely accurate: they are rather the path of the evolutionary progress that is repeatedly explored by life, at all times, since Lamarck believed in spontaneous generation. His trees are certainly polarized in time in the sense that the highest form in the trees has to appear before the lowest ones, as the evolution progresses. However each form in the tree is also an evolutionary/developmental stage at which some creatures stay (when they do not keep evolving). The chronology is thus messed up in these drawings. For instance, there are both recent and ancient polyps, some of which are then more recent than some of the Ascidians, although the Ascidians as a form are "lower" than the polyps on the tree.

Author's response

In the paragraph in question, I introduce Lamarck's evolutionary theory by stating that "Lamarck made evolutionary change, including the ongoing spontaneous creation of primitive forms and the upward transformation of existing life, the centrepiece of his evolutionary theory." His trees are based on temporal progression, but not of a kind familiar to us post-Darwinians. I have now re-worded this sentence to read: "The titles of these trees reveal their evolutionary intent...".

Reviewer comments

The section "Trees before Darwin" should be entitled "Biological trees before Darwin", because it does not comment, for instance, on Porphyry's most important trees in philosophy. Likewise, in the conclusion, the author should recall that the trees he introduced are presented in the historical context of *biology*. Otherwise, he would have had to comment on the most interesting fact that, at the very same date than Darwin published his famous tree, outside the field of biology, in linguistics and philology, a most famous tree of languages was also published by August Schleicher.

Author's response

Medieval illustrated manuscripts depict various dichotomies and *arbores *representing concepts *e.g*. from logic (notably the "tree of Porphyry" based on his *Isagoge*, itself a commentary on Aristotle's *Categoriae *and *De Interpretatione*), genealogy (a famous one is attributed to Isidore of Seville, *ca *560 - 636 AD) and mysticism (the Sephirotic tree, actually a network) [182,183]. The Reviewer rightly reminds us too of Schleicher's *Stammbaumtheorie*, depicted as a tree in publications beginning in 1853 [for a reproduction see [184], p.46]. August Schleicher, a professor at Jena, read Darwin's *Origin *on his colleague Ernst Haeckel's advice, and subsequently wrote articles presenting the origin and development of languages as a validation of Darwin's theory.

Reviewer comments

What does "emanationist" mean? Could the author describe this notion a bit more? Also, what does "osculant" mean?

Author's response

*Emanationist *describes unitary philosophical or cosmological systems according to which all that exists (the universe and everything within it) has arisen through a process of flowing-out from, and willed by, a deity or First Principle. This flowing-out necessarily gives rise to a hierarchy or continuum of entities of which those closest to the First Principle are the most-perfect, while those farther away are increasingly material, embodied and imperfect. These systems are to be contrasted with those positing a (perfect) creator who stands outside his (less-perfect) creation.

In the quinarian system, an *osculant *taxon is one positioned at the tangent between two large groups (circles of five taxa), sharing some characteristics with each. *Ancipiti natura hoc genus est, ambigens*.

### Reviewer's report 2

Patrick Forterre, Université Paris Sud and Institut Pasteur, Paris, France

Reviewer comments

The history of the metaphors used by biologists to depict relationships between organisms is a fascinating story that Mark Ragan presents in this paper in a lively and exhaustive way. I discovered in reading his paper that the metaphor of networks, so fashionable right now among evolutionists fascinated by lateral gene transfers, indeed predated the tree metaphor in pre-Darwinian times. Unfortunately, there is a great confusion in the use of the network metaphor right now. The hereditary history of living organisms can be depicted with a tree-like structure as long as new organisms originate by cell division. This seems to be the rule for most living organisms, since examples of fusion that prevent us from identifying the continuity of cellular lineages are rare. For instance plants are clearly a eukaryotic lineage that can be inserted into a eukaryotic tree, and not a peculiar lineage of cyanobacteria. Similarly, mammals are clearly a branch in the tree of animals, and not a peculiar form of retrovirus despite the fact that retroviruses and derived element comprise up to 80% of their genomes. It was one of the great successes of science in the XIX century to realize that organisms are related to each other *via *a tree and not a network. This was achieved thanks to progress in evolutionary theory and it's not a coincidence that the only illustration of the origin of species is precisely such a tree. The merit of the historical approach as depicted in Ragan's paper is to remind us that this tree did not come from nowhere but had to fight its way out of various pre-scientific networks. These networks were designed to take into account various observations that were not understood at the time, such as the existence of homologous characters in organisms from diverse lineages. We know now that these homologies are produced by common descent, by gene transfer between lineages, or by convergence, different processes that were combined in misleading ways in these old networks. Networks are again fashionable because organisms are now frequently confused with their genes and/or genomes (see a recent excellent review by Gribaldo and Brochier-Armant [185] on this issue). Genomes as integrated entities and genes taken independently evolve in a tree-like fashion (DNA replication produces tree-like heredity) but genomes are composed of genes whose history can vary from one gene to the other. A network is indeed a good metaphor to describe the movement of genes between genomes across the tree of organisms. This is especially true for genes originally encoded by viruses and their derivatives, plasmids, transposons and integrons. In that case, entire genomes from different organisms (virus-virus, virus-plasmids or plasmid-plasmid) can recombine to produce new lineages, which can be assimilated to a fusion. The evolutionary process is therefore a combination of tree-like processes (the evolution of cellular lineages from LUCA until now, the evolution of genes in general, the evolution of viral/plasmids lineages during much part of their history) and of network-like processes (the movement of genes between lineages, the formation of new plasmid/virus lineage by recombination). From this account by Mark Ragan, it seems that this dual nature of the evolutionary process has never been taken into account in the history of biology and that the tree and network metaphors were always considered to be in opposition. This may derive from the difficulty for most scientists of adopting a dialectic view of nature (evolution is both trees and networks) and their propensity to adopt a mechanistic approach (either/or) that favours opposition (between metaphors and, as a consequence, between scientists, who favour different metaphors!). Both historical and philosophical approaches may be required now to get rid of these false oppositions.

Reviewer comments

I thank the Reviewer for these insightful comments.

### Reviewer's report 3

Dan Graur, University of Houston, Houston, Texas, United States

Reviewer comments

This Reviewer provided no comments for publication. The author is grateful to the Reviewer and made a number of changes to the manuscript, particularly the final section, based on points he raised.

## Competing interests

The author declares that they have no competing interests.

## Authors' contributions

MAR planned and wrote this article, and unless otherwise indicated is responsible for the translations from French, Italian, Latin, German and Russian.

## References

[B1] Nieremberg IE Historia naturae, maxime peregrinae, libris xvi Antverpiae: Plantiniana Balthasaris Moreti 1635

[B2] Budge EAW The Book of the Dead. The Papyrus of Ani in the British Museum 1895 London: The British Museum lxx lxxi

[B3] Kirchweger AJ Aurea Catena Homeri Frankfurt and Leipzig: Johann Georg Böhme 1723

[B4] Lovejoy AO The Great Chain of Being. A Study of the History of an Idea 1965 New York: Harper originally published 1936

[B5] Kuntz ML Kuntz PG Eds Jacob's Ladder and the Tree of Life. Concepts of Hierarchy and the Great Chain of Being (revised edition). American University Studies, Series V. Philosophy 1987 14 New York: Peter Lang

[B6] *Sefer ha-Bahir *attributed to Rabbi Nehunia ben haKana http://www.nasori.org/sepher_ha-bahir.htm

[B7] de Bouelles C Physicorum elementorum Caroli Bouilli Samarobrini veromandui libri decem Paris: Ioannis Pari & Iodoci Badii Ascensii 1512

[B8] Llull R Liber de ascensu et decensu intellectus (1304) Raymundi Lully Doctoris illuminati de nova logica de correlativis necnon de ascensu et descensu intellectus: quibus siquide tribus libellis p Valencia: Jorge Costilla 1512

[B9] Aristotle Historia animalium [Ross WD (Series Editor): The Works of Aristotle] 1910 IV Oxford: Clarendon Press; Clarendon Press, Oxford

[B10] Wotton E De differentiis animalium libri decem Lutetiae Parisiorum: Vascosanum 1552

[B11] Caesalpino A De plantis libri xvi Florentiae: Georgium Marescottum 1583

[B12] Zaluziansky à Zaluzian A Methodi herbariae libri tres 1940 Pragae: Georgij Decziceni; 1592. Reprinted in part by Academiae Scientiarum et Artium Bohemicae, Pragae

[B13] Gesner C Historia animalium Tiguri [Zürich]: Christ. Froschoverum 1551 1587

[B14] de Lamarck JBPA Flore Françoise ou Description Succincte des Toutes les Plantes qui Croissant Naturellement en France, Disposée selon une Nouvelle Méthode d'Analyse... Tome Premier 1778 Paris: Imprimerie Royale

[B15] Stevens PF Haüy and A.-P. Candolle: crystallography, botanical systematics, and comparative morphology, 1780-1840 J Hist Biol 1984 17 49 82 10.1007/BF00397502

[B16] Bradley R A Philosophical Account of the Works of Nature London: Mears 1721

[B17] Dobell C Antony van Leeuwenhoek and his "Little Animals". Being some Account of the Father of Protozoology and Bacteriology and his Mutifarious Discoveries in these Disciplines. Collected, Translated, and Edited from his Printed Works, Unpublished Manuscripts, and Contemporary Records 1932 London: Staples Press

[B18] Boerhaave H Ed Bybel der Natuur, door Jan Swammerdam, Amsterdammer of Historie der Insecten Leyden: I Severinus, B Vander Aa, P Vander Aa 1737 1738

[B19] Seba A Locupletissimi rerum naturalium thesauri accurate descriptio, et iconibus artificiossissimis expressio, per universam physics historiam Amsterlaedami: Janssonio-Waesberrgios, J Wetstenium & Gul. Smith 1734

[B20] Renard L Poissons Ecrevisses et Crabes, de diverses Couleurs et Figures Extraordinaires, que l'on Trouve Autour des Isles Moluques, et sur les Côtes des Terres Australes Amsterdam: Reinier & Josué Ottens 1754

[B21] von Linné C Caroli Linnæi...Systema Naturae per Regna tria Naturae, secundum classes, ordines, genera, species, cum characteribus, differentiis, synonymis, locis..., Editio decima, reformata Holmiæ [Stockholm]: Laurentii Salvii 1758 1759

[B22] Greene EL Egerton FN Landmarks of Botanical History 1983 Stanford CA: Stanford University Press Part I first published 1909

[B23] Voltaire [Arouet F-M] Chaîne des êtres créés Dictionnaire Philosophique, portatif 1764 Londres: [no publisher recorded] 71 73

[B24] Johnson S A review of [Soame Jenyns's] A Free Enquiry into the Nature and Origin of Evil Lit Mag Univ Rev 1757 2 13-15

[B25] Eldredge N Gould SJ Schopf TJM Punctuated equilibria: an alternative to phyletic gradualism Models in Paleobiology 1972 San Francisco: Freeman, Cooper & Co 82 115

[B26] Lombard Peter Sententiarum libri quatuor I.44.1-2 (about 1150 AD) http://www.franciscan-archive.org/lombardus/opera/ls1-44.html

[B27] Ray J The Wisdom of God Manifested in the Works of the Creation..., corrected Seven London: R. Harbin 1717

[B28] Marsigli LF Histoire physique de la mer, ouvrage enrichi de figures dessinées d'après le naturel Amsterdam: de la Compaigne 1725

[B29] Peyssonel JA New observations upon the worms that form sponges. Translated from the French Phil Trans 1758 50 590 594 10.1098/rstl.1757.0079

[B30] Ellis J An essay towards a Natural History of the Corallines, and other Marine Productions of the like Kind, Commonly found On the Coasts of Great Britain and Ireland London: printed for the author 1755

[B31] Trembley A Mémoires, pour servir à l'Histoire d'un Genre de Polypes d'Eau douce, à bras en Forme de Cornes Leide: Jean & Herman Verbeek 1744

[B32] Rösel von Rosenhof AJ Der monatlich-herausgegebenen Insecten-Belüstigüng Nurnberg: Johann Joseph Fleischmann 1746 1763

[B33] Bonnet C Contemplation de la Nature. Amsterdam: Marc-Michel Rey; 1764. Pages cited are from the octavo Oeuvres d'Histoire naturelle et de Philosophie de Ch. Bonnet, Tome VII Neuchâtel: Fauche 1781

[B34] Linnaeus C Philosophia botanica in qua explicantur fundamenta botanica cum definitionibus partium, exemplis terminorum, observationibus rariorum, adjectus figuris aeneis Stockholmiae: Godofr Kiesewetter, & Amstelodami: Z. Chatelain 1751

[B35] Flittner CG Gynäologie, Bdchn X. Die Begattung und Fortpflanzung organischer Wesen nach der Stufenleiter der Natur Berlin: Oehmigke 1797

[B36] Ragan MA A third kingdom of eukaryotic life: history of an idea Arch Protistenkd 1997 148 225 243

[B37] Ragan MA On the delineation and higher-level classification of algae Eur J Phycol 1998 33 1 15 10.1080/09670269810001736483

[B38] Ritterbush PC Overtures to Biology. The Speculations of Eighteenth Century Naturalists 1964 New Haven: Yale University Press

[B39] Raio [Ray] J Methodus plantarum nova, brevitatis & perspicuitatis causa synoptice in tabulis exhibita; cum notis generum tum summorum tum subalternorum characteristicis, observationibus nonnullis de seminibus plantarum & indice copioso Londini: Henrici Faitborne & Joannis Kersey; 1682. Quotation is in Præfatio ad Lectorem, second page (unnumbered)

[B40] Linné C Praelectiones in ordines naturales plantarum. E priprio et Jo. Chr. Fabricii...edidit Paulus Diet. Giseke...Accessit uberior palmarum et scitaminum expositio praeter plurium novorum generum reductiones cum mappa geographico-genealogica affinitatum ordinum, et aliquot fructuum palmarum figurae Hamburgi: Benj. Gottl. Hoffmanni 1792

[B41] de Candolle A Troisième mémoire sur la famille des Myrsinéacées Ann Sci Nat Bot (sér 2) 1841 16 148 168

[B42] von Sachs J History of Botany (1530-1860). Translation by HEF Garnsey; revised by IB Balfour 1890 New York: Russell & Russell

[B43] Stevens PF Augustin Augier's "Arbre botanique" (1801), a remarkable early botanical representation of the natural system Taxon 1983 32 203 211 10.2307/1221972

[B44] O'Hara RJ Representations of the Natural System in the Nineteenth Century Biol Philos 1991 6 255 274 10.1007/BF02426840

[B45] Stevens PF The Development of Biological Systematics. Antoine-Laurent de Jussieu, Nature, and the Natural System 1994 New York: Columbia University Press

[B46] Whewell W The Philosophy of the Inductive Sciences founded upon their History, New Edition 1847 London: John W. Parker

[B47] Balfour JH Botany Encyclopaedia Britannica 1876 4 Nine 79 163

[B48] Pallas PS Elenchus zoophytorum sistens generum adumbrationes generaliores et specierum cognitarum succinctas descriptiones cum selectis auctorum synonymis Hagae-Comitum [The Hague]: Petrum van Cleef 1766

[B49] Pallas PS Charakteristik der Thierpflanzen, worin von den Gattungen derselben allgemeine Entwürse, und von denen dazugehörigen Arten kurze Beschreibungen gegeben werden; nebst den vornehmsten Synonymen der Schriftsteller Nürnberg: Kaspischen Buchhandlung 1787

[B50] Thienemann A Die Stufenfolge der Dinge, der Versuch eines natürlichen Systems der Naturkörper aus dem achtenzehnten Jahrhundert. Eine historische Skizze Zoologische Annalen (Würzburg) 1910 3 185 274

[B51] Eichwald E Zoologia specialis quam expositis animalibus tum vivis, tum fossilibus potissimum Rossiae in universum, et Poloniae in species, in usum lectionum publicarum in Universitate Caesarea Vilnensi habendarum. Pars prior. Propaedeuticam zoologiae atque specialem Heterozoorum expositionem continens 1829 Vilnae: Josephus Zawadzki

[B52] Mikulinskii SR Ed Istoriya Biologii s Drevneishiky Vremen do Nachala XX Veka [History of Biology from Ancient Times to the Beginning of the Twentieth Century] 1972 Moscow: Nauka

[B53] Mikulinskii SR Razvitie Obshchikh Problem Biologii v Rossii. Pervaya Polovina XIX Veka [Development of General Problems of Biology in Russia. First Half of the 19th Century] 1961 Moscow: USSR Academy of Science

[B54] Eichwald E De regni animalis limitibus atque evolutionis gradibus. Specimen quod consentiente amplissimo philosophorum ordine Univers. Caes. Dorpat. Ut veniam legendi rite sibi acquibat mens. Octobr. Publicae disceptationi submittit 1821 Dorpat: Joannis Christian Schünmann

[B55] Augier A Essai d'une nouvelle Classification des Végétaux conforme à l'Ordre que la Nature paroit avoir suivi dans le Règne Végétal: d'ou Resulte une Méthode qui conduit à la Conaissance des Plantes & de leur Rapports naturels 1801 Lyon: Bruyset Ainé

[B56] Lamarck J-B Recherches sur l'Organisation des Corps Vivans et particulièrement sur son Origine, sur la Cause de ses Développemens et des Progrès de sa Composition, et sur celle qui, tendant continuellement à la Détruire dans chaque Individu, amène nécessairement sa Mort; Précédé du Discours d'Ouverture du Cours de Zoologie, donné dans le Muséum national d'Histoire Naturelle 1802 Paris: Maillard

[B57] Corsi P The Age of Lamarck. Evolutionary Theories in France 1790-1830 1988 Berkeley: University of California Press

[B58] Lamarck JBPA Philosophie Zoologique, ou Exposition des Considérations relatives à l'Histoire naturelle des Animaux; à la Diversité de leur Organisation et des Facultés qu'ils en obtiennent; aux Causes physiques qui Maintiennent en eux la Vie et donnent lieu aux Mouvemens qu'ils exécutent; enfin, à celles qui produisent, les Unes le Sentiment, et les autres l'Intelligence de ceux qui en sont Doués 1809 Paris: Dentu, et l'Auteur

[B59] Lamarck J-B Histoire Naturelle des Animaux sans Vertèbres, prèsentant les Caractères Généraux et Particuliers de ces Animaux, leur Distribution, leurs Classes, leurs Familles, leurs Genres, et la Citation des principales Espèces qui s'y Rapportent; Précédée d'une Introduction offrant la Détermination des Caractères essentiels de l'Animal, sa Distinction du Végétal et des autre Corps naturels, enfin, l'Exposition des Principes Fondamentaux de la Zoologie. Tome Premier 1815 Paris: Verdière

[B60] Appel TA The Cuvier - Geoffroy Debate. French Biology in the Decades before Darwin 1987 New York: Oxford University Press

[B61] Burkhardt RW Jr The Spirit of System. Lamarck and Evolutionary Biology 1995 Cambridge MA: Harvard University Press

[B62] Donati V Della Storia Naturale Marina dell'Adriatico 1750 Venezia: Francesco Storti

[B63] Donati V Essai sur l'Histoire Naturelle de la Mer Adriatique, avec une lettre du Docteur Leonard Sesler, sur une Nouvelle Espece de Plante Terrestre 's-Gravenhage [The Hague]: Pierre de Hondt 1758

[B64] Olivi G Zoologia Adriatica, ossia Catalogo ragionato degli Animali del Golfo e delle Lagune di Venezia Bassano: G. Rovatii 1792

[B65] Treviranus GR Biologie, oder Philosophie der lebenden Natur für Naturforscher und Aerzte Göttingen: Röwer 1802 1818

[B66] Rühling IoP Ordines naturales plantarum commentatio botanica Goettingae: Abrah. Vandenhoeck 1774

[B67] Hermann J Tabula affinitatum animalium olim academico specimine edita 1783 Argentorati [Strasbourg]: Joh. Georgii Treuttel

[B68] Batsch AJGC Tabula affinitatum regni vegetabilis, quam delineavit, et nunc ulterius adumbratam 1802 Vinariae [Weimar]: Landes-Industrie-Comptior

[B69] Cuvier G Valenciennes A Histoire Naturelle des Poissons, Tome Premier Paris: F.G. Levrault 1828 1849

[B70] de Buffon GLL Daubenton LJM Histoire Naturelle... avec la Description du Cabinet du Roi. Tom IV. Quadrupeds Paris: Imprimerie Royale 1753

[B71] Goldfuss GA Ueber die Entwicklungsstufen des Thieres 1817 Nürnberg: L. Schrag

[B72] de Jussieu AHL Sur le groupe de Rutacées Mém Mus Hist Nat 1825 12 384 542

[B73] Horaninow P Primae lineae systematis naturae, nexui naturali omnium evolutionique progressivae per nixus reascendentes superstructui 1834 Petropoli: Krajanis

[B74] Swainson W A preliminary discourse on the study of natural history. Lardner's Cabinet Cyclopædia 1834 59 London: Longman, Rees, Orme, Brown, Green & Longman

[B75] Fischer de Waldheim G Tableaux synoptiques de Zoognosie, publiés à l'Usage de ses Élèves à l'Université Imperiale de Moscou 1805 Moscou: Université

[B76] Macleay WS Horae entomologicae: or Essays on the Annulose Animals. (Parts I and II) I London: S. Bagster 1819 1821

[B77] Swainson WJ On the Natural History and Classification of Birds 1837 2 London: Longman, Rees, Orme, Brown, Green, and Longman

[B78] Milne-Edwards [H] Considérations sur quelques principes relatifs a la classification naturelle des animaux, et plus particulièrement sur la distribution méthodique des mammifères Ann Sci Nat 3 me sér 1844 1 65 99

[B79] Vigors NA Observations on the natural affinities that connect the orders and families of birds Trans Linn Soc 1824 14 395 517 10.1111/j.1095-8339.1823.tb00098.x

[B80] Lindley J Exogens Penny Cyclopædia of the Society for the Diffusion of Useful Knowledge 1838 10 London: Charles Knight & Co 120 131 http://books.google.com

[B81] Coggin J Quinarianism after Darwin's *Origin*: the circular system of William Hincks J Hist Biol 2002 35 5 42 10.1023/A:1014582710287

[B82] Kaup JJ Einige Worte über die systematische Stellung der Familie der Raben, Corvidae J Ornithol 1854 2 Jahresversammlung xlvii lvii

[B83] Newman E Further observations on the septenary system Entomolog Mag 1837 4 234 251

[B84] Fries EM Systema orbis vegetabilis primas lineas novæ constructionis .. Pars 1. Plantae homonemeæ 1825 Lund: Typographica Academia

[B85] Strickland HE On the true method of describing the natural system in zoology and botany Ann Mag Nat Hist 1841 6 184 194

[B86] Anonymous [Chambers R] Vestiges of the Natural History of Creation 1844 London: John Churchill

[B87] Miller H The Testimony of the Rocks; or, Geology in its Bearings on the Two Theologies, Natural and Revealed 1857 Boston: Gould & Lincoln

[B88] Darwin CR Letter 814, Darwin CR to Hooker JD 1845 http://www.darwinproject.ac.uk/darwinletters/calendar/entry-814.html

[B89] Ruse M Monad to Man. The Concept of Progress in Evolutionary Biology 1996 Cambridge MA: Harvard University Press

[B90] Hitchcock E Elementary Geology 1840 Amherst: J.S. & C. Adams

[B91] Archibald JD Edward Hitchcock's pre-Darwinian (1840) "Tree of Life" J Hist Biol 2009 42 561 592 10.1007/s10739-008-9163-y 20027787

[B92] Agassiz L Recherches sur les Poissons Fossiles. Tome I. Contenant l'Introduction et toutes les Questions Générales, Anatomiques, Zoologiques et Géologiques Neuchatel: Petitpierre et Prince 1833 1845

[B93] Wallace AR On the law which has regulated the introduction of new species Ann Mag Nat Hist (n.s.) 1855 16 184 196

[B94] Wallace AR Attempts at a natural arrangement of birds Ann Mag Nat Hist (n.s.) 1856 18 193 219

[B95] Bronn HG Untersuchungen über die Entwickelungs-Gesetze der organischen Welt während der Bildungs-Zeit unserer Erd-Oberfläche 1858 Stuttgart: E. Schwiezerbart'sche Verlagshandlung und Druckerei

[B96] Nyhart LK Biology takes Form. Animal Morphology and the German Universities, 1800-1900 1995 Chicago: University of Chicago Press

[B97] Gliboff S H.G. Bronn, Ernst Haeckel, and the Origins of German Darwinism. A Study in Translation and Transformation 2008 Cambridge MA: MIT Press

[B98] Barrett PH Gautrey PJ Herbert S Kohn D Smith S Eds Charles Darwin's Notebooks, 1836-1844 1987 London: British Museum (Natural History)

[B99] Bredekamp H Darwins Korallen: Frühe Evolutionsmodelle und die Tradition der Naturgeschichte 2005 Berlin: Klaus Wagenbach Verlag

[B100] Voss J Darwins Bilder. Ansichten der Evolutionstheorie 1827 bis 1874 2007 Frankfurt am Main: Fischer Taschenbuch Verlag

[B101] Kirchhoff G Ueber die Auflösung der Gleichungen, auf welche man bei der Untersuchung der linearen Vertheilung galvanischer Ströme gefürt wird Ann Phys Chem 1847 72 497 508 10.1002/andp.18471481202

[B102] von Staudt KGC Geometrie der Lage 1847 Nürnberg: Verlag von Bauer und Raspe (Julius Merz)

[B103] Cayley A On the theory of analytical forms called trees Lond Edinb Dubl Phil Mag [ser. 4] 1857 13 172 176

[B104] Darwin CR On the Origin of Species by Means of Natural Selection, or the Preservation of Favoured Races in the Struggle for Life 1859 1 1 London: John Murray PMC5184128 30164232

[B105] Dennett DC Darwin's Dangerous Idea. Evolution and the Meanings of Life 1995 New York: Touchstone Books/Simon & Schuster

[B106] Haeckel E Generelle Morphologie der Organismen. Allgemeine Grundzüge der organischen Formen-Wissenschaft mechanisch begründet durch die von Charles Darwin reformirte Decendenz-Theorie 1866 Berlin: Reimer

[B107] Haeckel E Anthropogenie oder Entwickelungsgeschichte des Menschen. Gemeinverständliche wissenschaftliche Vorträge über die Grundzüge der menschlichen Keimes- und Stammes-Geschichte 1874 Leipzig: Engelmann

[B108] Dayrat B The roots of phylogeny: how did Haeckel build his trees? Syst Biol 2003 52 515 527 12857642 10.1080/10635150390218277

[B109] Kusnezov NI Subgenus Eugentiana Kusnez. generis *Gentiana *Tournef Acta Horti Petropolitani 1894 15 1 507

[B110] Ragan MA McInerney JO Lake JA The network of life: genome beginnings and evolution Phil Trans R Soc Lond B: Biol Sci 2009 364 2169 2185 10.1098/rstb.2009.0046 PMC2874017 19571237

[B111] Garrod AH On some points in the anatomy of the parrots which bear on the classification of the suborder Proc Zool Soc Lond 1874 1874 586 598

[B112] Sharpe RB A review of recent attempts to classify birds: an address delivered before the Second International Ornithological Congress on the 18th of May, 1891 1891 Budapest: Hungarian National Museum http://www.archive.org/details/reviewofrecentat00shar

[B113] O'Hara RJ Ouellet H Diagrammatic classifications of birds, 1819-1901: views of the Natural System in 19^th^-Century British ornithology Acta XIX Congressus Internationalis Ornithologici: 22-29 June 1986; Ottawa 1988 II Ottawa: National Museum of Natural Sciences 2746 2759

[B114] Lam HJ Phylogenetic symbols, past and present (Being an apology for genealogical trees) Acta Biotheoretica 1936 2 153 194 10.1007/BF01556309

[B115] Linnaeus C Systema Naturae Lugduni Batavorum [Leiden]: T. Haak 1735

[B116] Leibniz GW Erdmann JE[untitled handwritten manuscript sometimes known as Principia Philosophiae seu Theses in Gratiam Principis Eugenii Conscriptae, after Deutens's 1721 Latin translation]; 1714 1840 Berlin: Eichler 705 712

[B117] Kützing FT Phycologia Generalis oder Anatomie, Physiologie und Systemkunde der Tange 1843 Leipzig: Brockhaus

[B118] Haeckel E Das Protistenreich. Eine populäre Uebersicht über das Formengebiet der niedersten Lebewesen. Mit einem wissenschaftlichen Anhange: System der Protisten 1878 Leipzig: Ernst Günther

[B119] Lamouroux JVF Essai sur les genres de la famille des Thalassiophytes non articulés Ann Mus (Nat) Hist Nat (Paris) 1813 20 21 47 115-139,267-293

[B120] Fritsch FE The Structure and Reproduction of the Algae. Introduction, Chlorophyceae, Xanthophyceae, Chrysophyceae, Bacillariophyceae, Cryptophyceae, Dinophyceae, Chloromonadophyceae, Euglenineae, Colourless Flagellata 1935 I Cambridge: Cambridge University Press

[B121] Tilden JE The Algae and their Life Relations. Fundamentals of Phycology 1937 Second Minneapolis: University of Minnesota Press

[B122] Magne F Classification et phylogénie des Rhodophycées Cryptogam Algol 1989 10 101 115

[B123] Silva PC Irvine DEG, John DM The role of extrinsic factors in the past and future of green algal systematics Systematics of the Green Algae. The Systematics Association Special 1984 27 London: Academic Press 419 433

[B124] McFadden GI Primary and secondary endosymbiosis and the origin of plastids J Phycol 2001 37 951 959 10.1046/j.1529-8817.2001.01126.x

[B125] Yoon HS Hackett JD Bhattacharya D A single origin of the peridinin- and fucoxanthin-containing plastids in dinoflagellates through tertiary endosymbiosis Proc Natl Acad Sci USA 2002 99 11724 11729 10.1073/pnas.172234799 12172008 PMC129336

[B126] Sommer MS Gould SB Kawach O Klemme C Voß C Maier U-G Zauner S Katz LA, Bhattacharya D Photosynthetic organelles and endosymbiosis Genomics and Evolution of Microbial Eukaryotes 2006 Oxford: Oxford University Press 94 108

[B127] Klebs G Flagellatenstudien. Theil II Zeitschr wiss Zool 1893 55 353 445

[B128] Gurney-Dixon S The Transmutation of Bacteria 1919 Cambridge: Cambridge University Press

[B129] Schmitt RM Zur Variabilität der Enteritis-bakterien Zeitschr Infektionskrankh parasit Krankh Hyg Haustiere 1911 9 188

[B130] Griffith F The significance of pneumococcal types J Hyg 1928 27 113 159 10.1017/S0022172400031879 PMC2167760 20474956

[B131] Avery OT MacLeod CM McCarty M Studies on the chemical nature of the substance Inducing transformation of pneumococcal types: induction of transformation by a desoxyribonucleic acid fraction Isolated from Pneumococcus Type III J Exp Med 1944 79 137 158 10.1084/jem.79.2.137 19871359 PMC2135445

[B132] Brüssow H The not so universal tree of life *or *the place of viruses in the living world Phil Trans R Soc Lond B: Biol Sci 2009 364 2263 2274 10.1098/rstb.2009.0036 PMC2873004 19571246

[B133] Stanier RY van Niel CB The main outlines of bacterial classification J Bacteriol 1941 42 437 466 16560462 10.1128/jb.42.4.437-466.1941 PMC374769

[B134] Winogradsky S Sur la classification des bactéries Ann Inst Pasteur 1952 82 125 131 14952929

[B135] van Niel CB Kessel EL Classification and taxonomy of the bacteria and blue green algae A Century of Progress in the Natural Sciences, 1853-1953 1955 San Francisco: California Academy of Sciences 89 114

[B136] Stanier RY Doudoroff M Adelberg EA The Microbial World 1957 Englewood Cliffs NJ: Prentice-Hall Inc

[B137] Olsen JG Woese CR Overbeek RJ The winds of (evolutionary) change: breathing new life into microbiology J Bacteriol 1994 176 1 6 8282683 10.1128/jb.176.1.1-6.1994 PMC205007

[B138] Woese CR There must be a prokaryote somewhere: microbiology's search for itself Microbiol Rev 1994 58 1 9 8177167 10.1128/mr.58.1.1-9.1994 PMC372949

[B139] Sapp J The prokaryote-eukaryote dichotomy: meanings and mythology Microbiol Mol Biol Revs 2005 69 292 305 10.1128/MMBR.69.2.292-305.2005 PMC1197417 15944457

[B140] Marmur J Falkow S Mandel M New approaches to bacterial taxonomy Ann Rev Microbiol 1963 17 329 372 10.1146/annurev.mi.17.100163.001553 14147455

[B141] McCarthy BJ Bolton ET An approach to the measurement of genetic relatedness among organisms Proc Natl Acad Sci USA 1963 50 156 164 10.1073/pnas.50.1.156 13932048 PMC300669

[B142] De Ley J Dobzhansky T, Hecht MK, Steere WC Molecular biology and bacterial phylogeny Evolutionary Biology 1968 2 New York: Appleton-Century Crofts 103 156

[B143] Zuckerkandl E Pauling L Molecules as documents of evolutionary history J Theor Biol 1965 8 357 366 10.1016/0022-5193(65)90083-4 5876245

[B144] Doolittle RF Blombach B Amino-acid sequence investigations of fibrinopeptides from various mammals: evolutionary implications Nature 1964 202 147 152 10.1038/202147a0 14156289

[B145] Edwards AWF Cavalli-Sforza LL Heywood VH, McNeill J Reconstruction of evolutionary trees Phenetic and Phylogenetic Classification: a Symposium. Systematics Association Publication 6 1964 London: Systematics Association 67 76

[B146] Eck RV Dayhoff MO Atlas of Protein Sequence and Structure 1966 Silver Spring MD: National Biomedical Research Foundation

[B147] Fitch WM Margoliash E Construction of phylogenetic trees: a method based on mutational distances as estimated from cytochrome *c *sequences is of general applicability Science 1967 155 279 284 10.1126/science.155.3760.279 5334057

[B148] Dayhoff MO Computer analysis of protein evolution Sci Amer 1969 221 1 87 95 10.1038/scientificamerican0769-86 5789703

[B149] Jardine N van Rijsbergen CJ Jardine CJ Evolutionary rates and the inference of evolutionary tree forms Nature 1969 224 185 10.1038/224185a0

[B150] Ambler RP Daniel M Hermoso J Meyer TE Bartsch RG Kamen MD Cytochrome c_2 _sequence variation among the recognised species of purple nonsulphur photosynthetic bacteria Nature 1979 278 659 660 10.1038/278659a0 221822

[B151] Ambler RP Meyer TE Kamen MD Anomalies in amino acid sequences of small cytochromes *c *and cytochromes *c' *from two species of purple photosynthetic bacteria Nature 1979 278 661 662 10.1038/278661a0 221823

[B152] Dickerson RE Evolution and gene transfer in purple photosynthetic bacteria Nature 1980 283 210 212 10.1038/283210a0 6243179

[B153] Woese CR Gibson J Fox GE Do genealogical patterns in purple photosynthetic bacteria reflect interspecific gene transfer? Nature 1980 283 212 214 10.1038/283212a0 6243180

[B154] Sogin SJ Sogin ML Woese CR Phylogenetic measurement in prokaryotes by primary structural characterization J Mol Evol 1972 1 173 184 10.1007/BF01659163 24173440

[B155] Fox GE Pechman KR Woese CR Comparative cataloging of 16S ribonucleic acid: molecular approach to prokaryotic systematics Int J Syst Bacteriol 1977 27 44 57

[B156] Woese CR Fox GE Phylogenetic structure of the prokaryotic domain: the primary kingdoms Proc Natl Acad Sci USA 1977 74 5088 5090 10.1073/pnas.74.11.5088 270744 PMC432104

[B157] Woese CR Bacterial evolution Microbiol Rev 1981 51 221 271 10.1128/mr.51.2.221-271.1987 PMC373105 2439888

[B158] Schwartz RM Dayhoff MO Origins of prokaryotes, eukaryotes, mitochondria, and chloroplasts Science 1978 199 395 403 10.1126/science.202030 202030

[B159] Woese CR Fox GE Zablen L Uchida T Bonen L Pechman K Lewis BJ Stahl D Conservation of primary structure in the 16S ribosomal RNA Nature 1975 254 83 86 10.1038/254083a0 1089909

[B160] Woese CR Fox GE Phylogenetic structure of the prokaryotic domain: the primary kingdoms Proc Natl Acad Sci USA 1977 74 5088 5090 10.1073/pnas.74.11.5088 270744 PMC432104

[B161] Fox GE Stackebrandt E Hespell RB Gibson J Maniloff J Dyer TA Wolfe RS Balch WE Tanner RS Magrum LJ Zablen LB Blakemore R Gupta R Bonen L Lewis BJ Stahl DA Luehrsen KR Chen KN Woese CR The phylogeny of prokaryotes Science 1980 209 457 463 10.1126/science.6771870 6771870

[B162] Pace NR Stahl DA Lane DJ Olsen GJ Analyzing natural microbial populations by rRNA sequences ASM News 1985 51 4 12

[B163] Rothschild LJ Ragan MA Coleman AW Heywood P Gerbi SA Are rRNA sequence comparisons the Rosetta Stone of phylogenetics? Cell 1986 47 640 10.1016/0092-8674(86)90505-2 3779840

[B164] Jain R Rivera MC Lake JA Horizontal gene transfer among genomes: the complexity hypothesis Proc Natl Acad Sci USA 1999 96 3801 3806 10.1073/pnas.96.7.3801 10097118 PMC22375

[B165] Beiko RG Harlow TJ Ragan MA Highways of gene sharing in prokaryotes Proc Natl Acad Sci USA 2005 102 14332 14337 10.1073/pnas.0504068102 16176988 PMC1242295

[B166] Dagan T Artz-Randrup Y Martin W Modular networks and cumulative impact of lateral transfer in prokaryote genome evolution Proc Natl Acad Sci USA 2008 105 10039 10044 10.1073/pnas.0800679105 18632554 PMC2474566

[B167] Dagan T Martin W Getting a better picture of microbial evolution en route to a network of genomes Phil Trans R Soc Lond B: Biol Sci 2009 364 2187 1296 10.1098/rstb.2009.0040 PMC2873007 19571239

[B168] Haggerty LS Martin FJ Fitzpatrick DA McInerney JO Gene and genome trees conflict at many levels Phil Trans R Soc Lond B: Biol Sci 2009 364 2209 2219 10.1098/rstb.2009.0042 PMC2873008 19571241

[B169] Soria-Carrasco V Castresana J Estimation of phylogenetic inconsistencies in the three domains of life Mol Biol Evol 2008 25 2319 2329 10.1093/molbev/msn176 18701430

[B170] Ragan MA Beiko RG Lateral genetic transfer: open issues Phil Trans R Soc Lond B: Biol Sci 2009 364 2241 2251 10.1098/rstb.2009.0031 PMC2872999 19571244

[B171] Doolittle WF Phylogenetic classification and the universal tree Science 1999 284 2124 2128 10.1126/science.284.5423.2124 10381871

[B172] Kunin V Goldovsky L Darzentas N Ouzounis CA The net of life: reconstructing the microbial phylogenetic network Genome Res 2005 15 954 959 10.1101/gr.3666505 15965028 PMC1172039

[B173] Chan CX Darling AE Beiko RG Ragan MA Are protein domains modules of lateral genetic transfer? PLoS ONE 2009 4 e4524 10.1371/journal.pone.0004524 19229333 PMC2639706

[B174] Wolf YI Rogozin IB Grishin NV Koonin EV Genome trees and the tree of life Trends Genet 2002 18 472 479 10.1016/S0168-9525(02)02744-0 12175808

[B175] Doolittle WF The practice of classification and the theory of evolution, and what the demise of Charles Darwin's tree of life hypothesis means for both of them Phil Trans R Soc Lond B: Biol Sci 2009 364 2221 2228 10.1098/rstb.2009.0032 PMC2873000 19571242

[B176] Dobzhansky T Biology, molecular and organismic Amer Zool 1964 4 443 452 10.1093/icb/4.4.443 14223586

[B177] Emmons P Embodying networks: bubble diagrams and the image of modern organicism J Architecture 2006 11 441 461 10.1080/13602360601037867

[B178] Sagan L On the origin of mitosing cells J Theor Biol 1967 14 225 274 10.1016/0022-5193(67)90079-3 11541392

[B179] Margulis L Origin of Eukaryotic Cells. Evidence and Research Implications for a Theory of the Origin and Evolution of Microbial, Plant, and Animal Cells on the Precambrian Earth 1970 New Haven: Yale University Press

[B180] Lloyd GER The development of Aristotle's theory of classification of animals Phronesis 1961 6 59 85 10.1163/156852861X00080

[B181] Pellegrin P Aristotle's Classification of Animals. Biology and the Conceptual Unity of the Aristotelian Corpus 1986 Berkeley: University of California Press

[B182] Murdoch JE Dichotomies and arbores Album of Science: Antiquity and the Middle Ages. [Cohen IB (Series Editor): Album of Science] 1984 I New York: Scribner 38 51

[B183] Cook R The Tree of Life. Image for the Cosmos 1974 New York: Avon

[B184] Williams DM Ebach MC Foundations of Systematics and Biogeography 2008 New York: Springer

[B185] Gribaldo S Brochier C Phylogeny of prokaryotes: does it exist and why should we care? Res Microbiol 2009 160 513 521 10.1016/j.resmic.2009.07.006 19631737

[B186] Woese CR Kandler O Wheelis ML Towards a natural system of organisms: proposal for the domains Archaea, Bacteria, and Eucarya Proc Natl Acad Sci USA 1990 87 4576 4579 10.1073/pnas.87.12.4576 2112744 PMC54159

